# Homotropic Cooperativity in Iron-Catalyzed Alkyne Cyclotrimerizations

**DOI:** 10.1021/acscatal.3c00764

**Published:** 2023-04-28

**Authors:** Ana M. Geer, Janeth Navarro, Pablo Alamán-Valtierra, Nathan T. Coles, Deborah L. Kays, Cristina Tejel

**Affiliations:** † Instituto de Síntesis Química y Catálisis Homogénea (ISQCH), Departamento de Química Inorgánica, Facultad de Ciencias, CSIC-Universidad de Zaragoza, Pedro Cerbuna 12, 50009 Zaragoza, Spain; ‡ School of Chemistry, University of Nottingham, University Park, Nottingham NG7 2RD, U.K.

**Keywords:** cooperativity, iron, alkynes, cyclotrimerization, kinetic studies

## Abstract

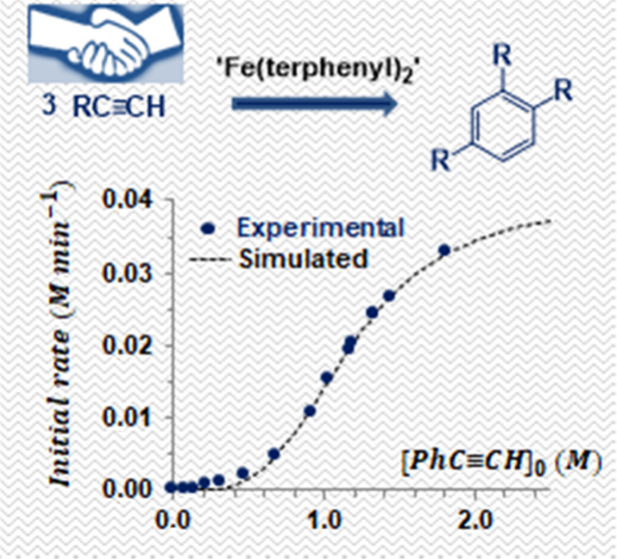

Enhancing catalytic activity through synergic effects is a current challenge in homogeneous catalysis. In addition to the well-established metal–metal and metal–ligand cooperation, we showcase here an example of self-*activation* by the substrate in controlling the catalytic activity of the two-coordinate iron complex [Fe(2,6-Xyl_2_C_6_H_3_)_2_] (**1**, Xyl = 2,6-Me_2_C_6_H_3_). This behavior was observed for aryl acetylenes in their regioselective cyclotrimerization to 1,2,4-(aryl)-benzenes. Two kinetically distinct regimes are observed dependent upon the substrate-to-catalyst ratio ([RC≡CH]_0_/[**1**]_0_), referred to as the *low* ([RC≡CH]_0_/[**1**]_0_ < 40) and *high* ([RC≡CH]_0_/[**1**]_0_ > 40) regimes. Both showed sigmoidal kinetic response, with positive Hill indices of 1.85 and 3.62, respectively, and nonlinear Lineweaver–Burk replots with an upward curvature, which supports positive substrate cooperativity. Moreover, two alkyne molecules participate in the *low* regime, whereas up to four are involved in the *high* regime. The second-order rate dependence on **1** indicates that binuclear complexes are the catalytically competent species in both regimes, with that in the *high* one being 6 times faster than that involved in the *low* one. Moreover, Eyring plot analyses revealed two different catalytic cycles, with a rate-determining step more endergonic in the *low* regime than in the *high* one, but with a more ordered transition state in the *high* regime than in the *low* one.

## Introduction

Cooperativity and synergistic effects have tremendous utility in the activation of small molecules and in homogeneous catalysis.^1^ Current interest focuses on the role of cooperating ligands in the synergistic activation of a substrate and in their behaviors as electron/proton reservoirs, which arises from reversible redox activity or easy aromatization/dearomatization processes, respectively.^2^ Besides this metal–ligand cooperation, polymetallic systems can benefit from metal–metal cooperation to activate one or two different substrates in a synergistic way, which is unattainable for the corresponding mononuclear species.^3^ Moreover, polymetallic catalysts showing redox cooperativity generally perform better at multielectron redox reactions.^4^ Recently, a combination of both metal–metal and metal–ligand cooperation has been observed in binuclear iron complexes.^5^

Another option involves the substrates behaving as the main cooperative actors.^6^ This approach is particularly well suited to many enzymes, such as diverse cytochrome P450 isoforms and phosphatases, and is also observed in organocatalysis.^7^ In this scenario, the binding of the first molecule of substrate increases the binding affinity of a second such that the catalytic activity is controlled by the concentration of the substrate in the reaction media. This self-activation by the substrate, also termed homotropic cooperativity, is characterized by kinetic patterns strongly deviated from the typical hyperbolic Michaelis–Menten curve and can be identified from kinetic studies.^6^

**Figure 1:**
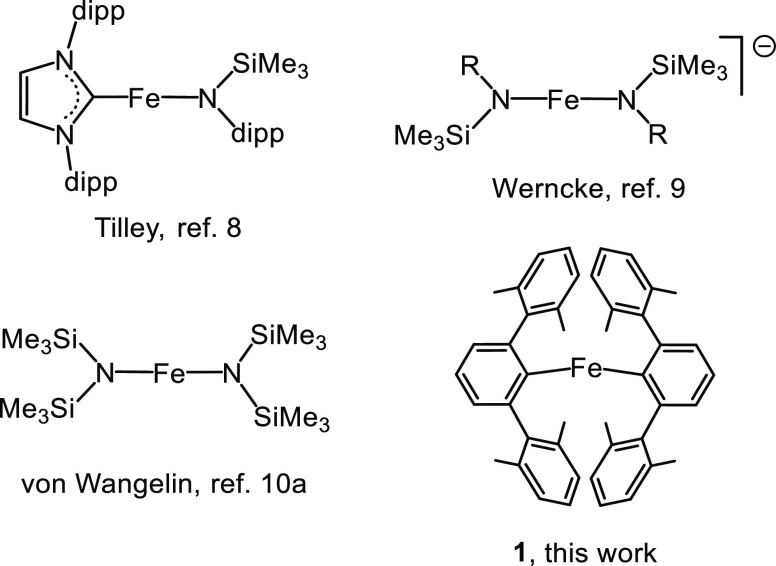
Two-coordinate iron complexes as catalysts for alkyne cyclotrimerization, R = Me_3_Si, dipp; dipp = 2,6-^*i*^Pr_2_C_6_H_3_.

Herein, we showcase an unprecedented case of homotropic cooperativity in the regioselective [2+2+2]-cycloaddition of acetylenes to 1,2,4-(aryl)benzenes catalyzed by a two-coordinate iron complex (**1**, Figure 1). The potential of such complexes in this field is poorly explored, with complexes [Fe(IPr){N(SiMe_3_)(dipp)}],^8^ [Fe{N(SiMe_3_)(R)}_2_]^−^,^9^ and [Fe{N(SiMe_3_)_2_}_2_],^10^ as the sole previously reported examples (Figure 1).

Related iron catalysts for this reaction^11^ include isolated low-valent iron(0) complexes such as tri-/tetra-coordinated complexes with diphosphines,^12^*N*-heterocyclic carbenes,^13^ diolefins,^14^ and pyrimidinediimine ligands.^15^ This last example exhibits an unusual 1,3,5-regioselectivity; typically the major product is the unsymmetrical 1,2,4-arene. In other instances, hydride iron complexes formed *in situ* such as [Fe^I^(pincer)(H)],^16^ and [Fe^II^(salen)(H)]^17^ have been proposed as the active species. Additionally, different combinations of iron salts and reductants^18^ or activators,^19^ as well as a three-component system (FeCl_2_/photoredox catalyst/reducing agent)^20^ also result in catalytically competent low-valent iron species.

## Results and Discussion

**Figure 2:**
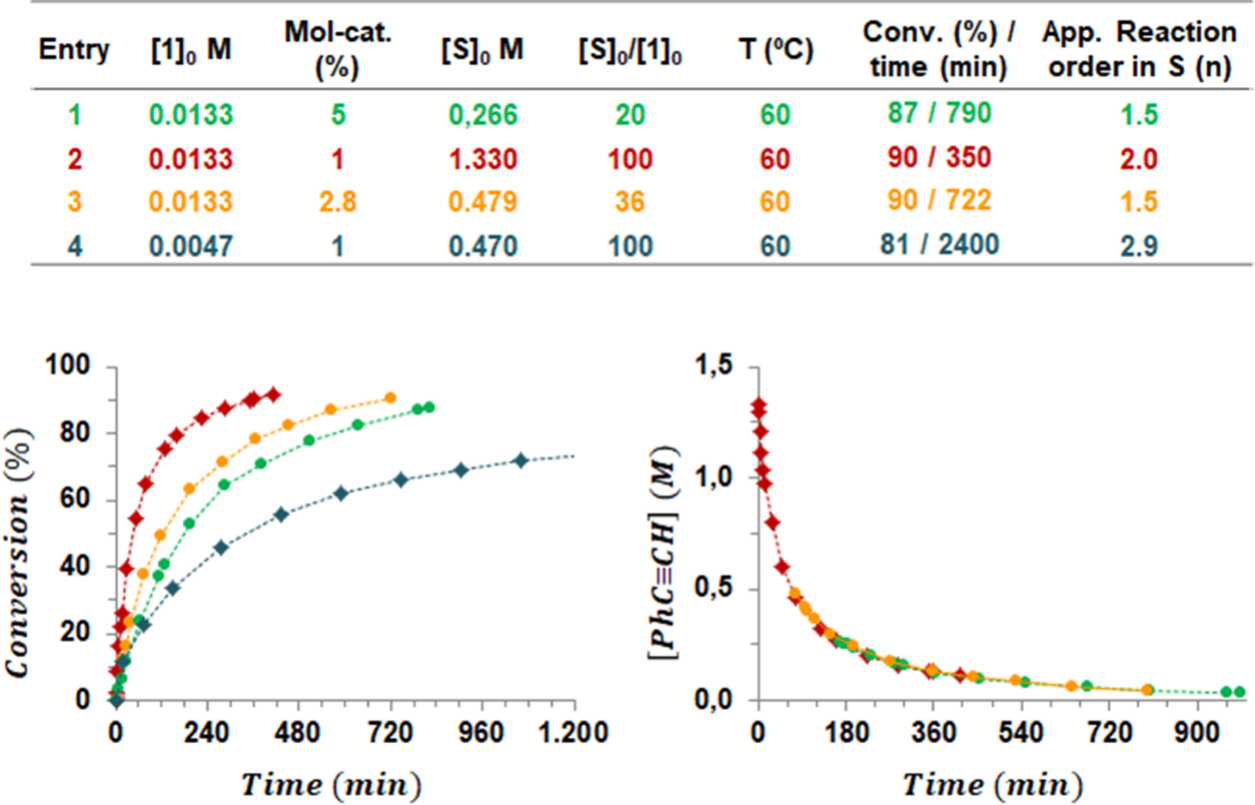
Conversion (%) *vs* time (min) plots (entries 1–4, left) and time-adjusted profiles (entries 1–3, right) for the cyclotrimerization of PhC≡CH (S) catalyzed by **1**. Experimental conditions and colors correspond to that shown in the figure. Dioxane (5 μL, 0.058 mmol) was used as internal standard. Dashed lines are for visual aid.

**Scheme 1:**
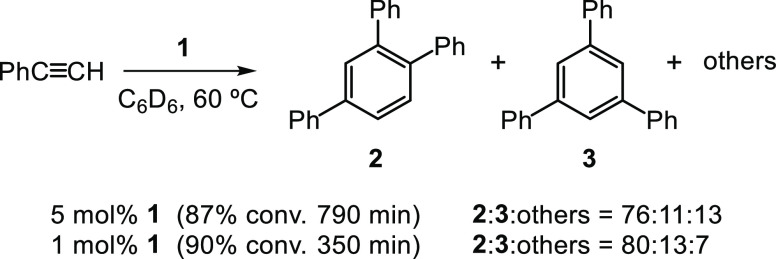
Phenylacetylene Cyclotrimerization Catalyzed by Complex **1**

An initial assessment for the catalytic activity of [Fe(2,6-Xyl_2_C_6_H_3_)_2_] (**1**, Xyl = 2,6-Me_2_C_6_H_3_, Figure 1)^21^ was performed with phenylacetylene (5 mol % **1**) in C_6_D_6_ at 60 °C (entry 1, Figure 2). The reaction was monitored by ^1^H NMR spectroscopy requiring around 13 h to achieve a good conversion (87%). The major product from the reaction was found to be the unsymmetrical 1,2,4-tri(phenyl)benzene (**2**) along with the symmetrical 1,3,5-tri(phenyl)benzene (**3**) and a small amount of unidentified oligomers with a ratio **2**:**3**:others of 76:11:13 (Scheme 1; Figure 2, entry 1).

A noticeable decrease in the reaction time was observed with a larger substrate loading and identical initial concentration of the precatalyst **1** (Figure 2, entry 2). Under these conditions, 90% of conversion takes place in around 6 h with slightly better yield and selectivity (ratio **2**:**3**:others of 80:13:7).

A particular feature of these two catalytic runs is their kinetic profiles, which were initially analyzed by using the general equation for an nth-order reaction for the substrate.^22^ The best fit for run 2 was obtained when plotting 1/[PhC≡CH] *versus* time, suggestive of a second-order reaction for the alkyne (Figure S3). Noticeably, an *apparent* fractional order (*n*) of 1.5 was obtained for run 1. A straight line required plotting 1/([PhC≡CH])^0.5^*versus* time (Figure S3).

This change in the *apparent* reaction order in PhC≡CH can be attributed to either the initial concentration of phenylacetylene or to the [PhC≡CH]_0_/[**1**]_0_ ratio. In order to discriminate between these, additional experiments at similar [PhC≡CH]_0_ but with [PhC≡CH]_0_/[**1**]_0_ ratios of 36 and 100 were carried out (Figure 2, entries 3 and 4, respectively). Run 3 showed again a good averaged fit to *n* = 1.5. However, the best fit for run 4 corresponds to an unusually *high* value of *n* = 2.9 (see below), which clearly indicates that the [PhC≡CH]_0_/[**1**]_0_ ratio plays a more important role than [PhC≡CH]_0_ (Figure S3).

Additional information was gathered from the time-adjusted profiles^23^ for runs 1–3 (with identical [**1**]_0_ and temperature), which were obtained by shifting the time scale of runs 3 and 1 to the point in which run 2 (with the highest substrate concentration) reaches [PhC≡CH] = 0.479 and 0.266 M, respectively. As shown in Figure 2 (right), once run 2 (in red) reaches a [PhC≡CH] = 0.479 M, it follows the same profile as run 3 (in orange). The same applies to runs 2 and 3 when reaching a [PhC≡CH] = 0.266 M that overlap with run 1. This excellent superposition clearly indicates that product inhibition and catalyst deactivation processes are negligible. In other words, evolution of the catalysis depends on the concentration of phenylacetylene in the reaction media (regardless of their initial concentration) and confirms that only the intrinsic kinetics contributes to the observed reaction rate.

**Figure 3:**
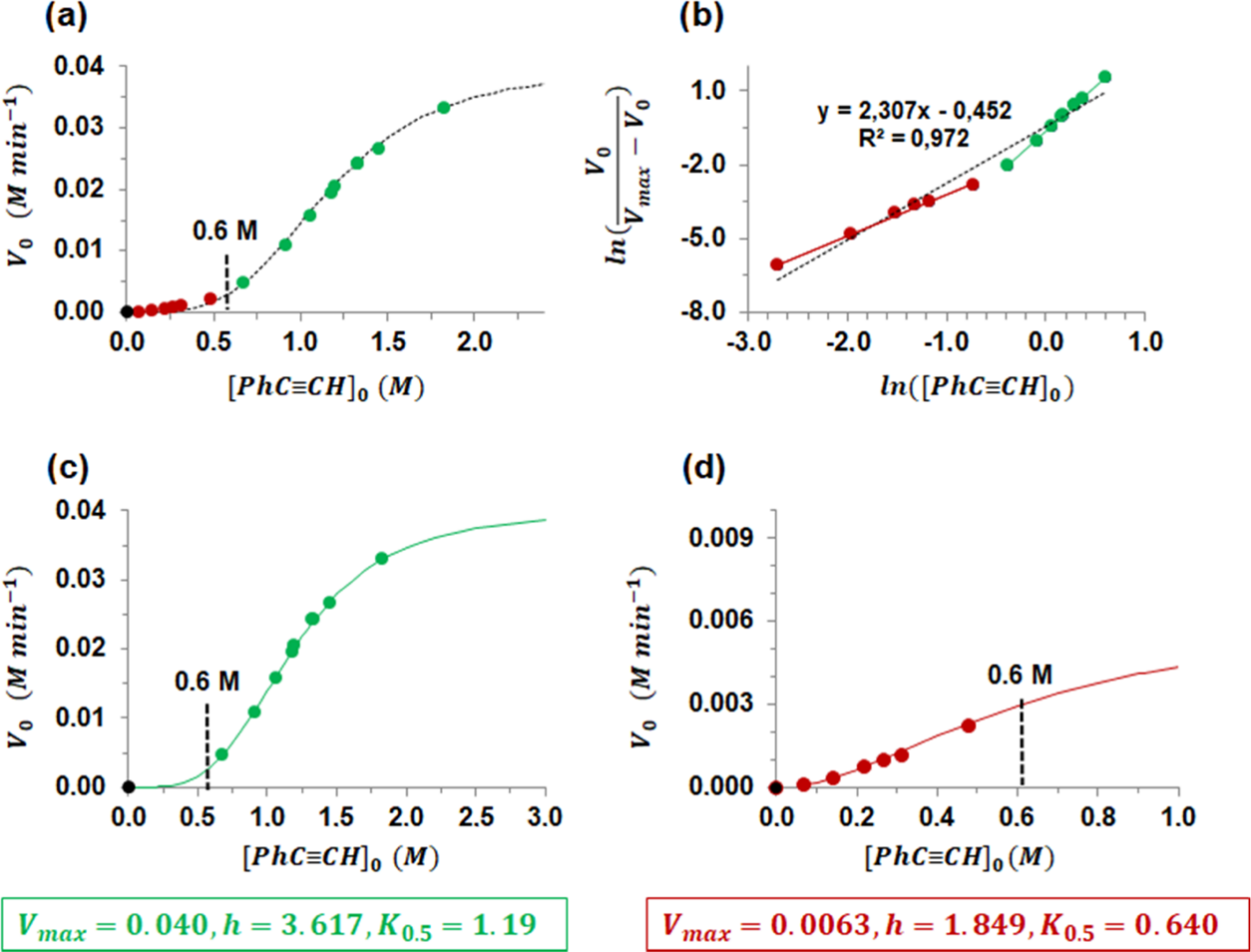
(a) Sigmoidal shape of the plot *V*_0_*vs* [PhC≡CH]_0_ and the simulated best fit (black line) for all of the experimental data: *V*_max_ = 0.040 ± 0.0016 M min^–1^, *K*_0.5_ = 1.17 ± 0.031 M, *h* = 3.62 ± 0.20 M. (b) Linear plot of the Hill equation clearly showing two straight lines for *V*_max_ = 0.040 M min^–1^. Experimental (circles) and simulated (lines) *V*_0_*vs* [PhC≡CH]_0_ plots for the *high* (c) and *low* (d) regimes, respectively. Parameters for the simulations of (c) and (d) are included in the green/red squares, respectively. Experimental conditions: [**1**]_0_ = 0.0133 M, *T* = 60 °C.

A second snapshot of this unusual behavior was attained by evaluating the dependence of the initial rate (*V*_0_) with the initial concentration of phenylacetylene at the same catalyst concentration ([**1**]_0_) (Table S1). Saturation kinetics with an increase of the initial concentration of phenylacetylene was observed, but, to our surprise, the curve displayed significant deviation from the hyperbolic Michaelis–Menten behavior, showing a sigmoidal shape. This feature implies that the overall catalytic process is cooperative in nature (Figure 3a).

The data were then analyzed using eq 1, where *V*_max_ refers to the maximum reaction rate, *K*_0.5_ is the substrate concentration that results in a reaction rate of 0.5(*V*_max_), and *h* (unitless), also known as the Hill index, reflects the level of cooperativity. Values of *h* > 1 indicate positive cooperativity, whereas *h* < 1 indicates negative cooperativity. Noncooperative processes correspond to *h* = 1, and eq 1 reduces to the Michaelis–Menten equation (eq 2).
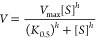
1
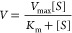
2

The major difference between the Michaelis–Menten behavior (eq 2) and those following eq 1 concerns the number of preequilibria previous to the rate-determining step that takes place, which is reduced to one in the latter case (cat + S ⇌ ′cat-S′ → Product).

The best fit^24^ for all of the data produced the following parameters: *V*_max_ = 0.040 ± 0.0016 M min^–1^, *K*_0.5_ = 1.17 ± 0.031 M, *h* = 3.62 ± 0.20, although it is not sufficient for [PhC≡CH] below 0.6 M (Figure 3a). Indeed, the linear plot of eq 1 (whose slope is *h*) for *V*_max_ = 0.040 gives an average value of *h* = 2.3 (Figure 3b, black line), quite far from the above-estimated value of 3.6. Interestingly, this linear plot revealed two well-defined regimes, namely, *low* (in red) and *high* (in green) (Figure 3b).

If we fit the data to the Hill equation for the *high* and *low* regimes independently, the plots in Figure 3c,d, respectively, are obtained. The value of *V*_max_ = 0.040 ± 0.0016 M min^–1^ for the *high* regime was directly obtained from the data. However, *V*_max_ for the *low* regime showed a large error (0.0078 ± 0.0039 M min^–1^) because of the lack of data at a *high* concentration of phenylacetylene in this regime. A more precise value of *V*_max_ for this regime of 0.0063 ± 0.00019 M min^–1^ was independently estimated by alternative methods (see below and Figure S1).

The positive Hill index (*h*) in both regimes indicates positive cooperativity such that coordination of one molecule of phenylacetylene to the catalyst facilitates the binding of the next, thus enhancing the catalytic activity. Moreover, analysis of the data using the Lineweaver–Burk replot showed the characteristic nonlinear upward curve for both regimes, again indicating positive cooperativity (Figure S2). The Hill index value of 1.85 in the *low* regime implies that cooperation is restricted to two molecules of phenylacetylene, which increases to four molecules at *high* [PhC≡CH]_0_/[**1**]_0_ ratio (*high* regime, *h* = 3.62).

For a catalytic system (**cat**) capable of interacting reversibly with up to two molecules of substrate (**S**) (eq 3), the Hill index reflects the relative values of the equilibrium constants *K*_1_ and *K*_2_
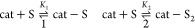
3such that positive cooperativy (*h* > 1) means that *K*_2_ > *K*_1_. If the system were “*infinitely*” cooperative (*K*_2_ ≫> *K*_1_) such that only species **cat** and **cat-S**_**2**_ are present in the reaction, a value of *h* = 2 is expected. Thus, the observed value of 1.85 in the *low* regime indicates a high level of cooperativity.

The same applies if four equilibria to **cat-S**_**4**_ are involved, where a value of *h* = 4 would reflect “*infinitely*” cooperative behavior involving four molecules of substrate and only the species **cat** and **cat-S**_**4**_ in the reaction media. Accordingly, the value of 3.62 estimated for the *high* regime is again indicative of a high level of cooperativity.

At this point, it is interesting to comment that, for kinetics following eq 1, *apparent* partial reaction orders in the substrate from zero to *h* can be observed depending on the relative values of *K*_0.5_ and [*S*]Scenario A:
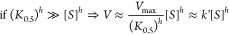
Scenario B:
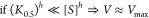


It is worth noting that the Hill indexes calculated represent the maximum partial reaction order in the substrate that could be observed in each regime. The observed rate order will be dependent upon the concentration of the substrate. For experiments in the *low* regime ([PhC≡CH]_0_/[**1**]_0_ < 40) and [**1**]_0_ ≈ 0.013 M, the data is represented closer to Scenario A, where the reaction is operating close to the maximum partial reaction order. Consequently, the observed partial reaction order in the alkyne of 1.50 (Figure 2, entries 1 and 3) matches well with the estimated value of *h* = 1.85.

For experiments in the *high* regime ([PhC≡CH]_0_/[**1**]_0_ = 100), two different *apparent* partial reaction orders in the alkyne were observed: 2 and 2.9 (entries 2 and 4, Figures 2 and S3, respectively). The former, which contains a [PhC≡CH]_0_ ≈ 1.33 M, is in the region between both Scenario A and B where (*K*_0.5_)^*h*^ is comparable to [*S*]^*h*^. This leads to a discrepancy of the partial reaction order of PhC≡CH to the maximum value of *h* (2 *vs* 3.82).

Lowering the [PhC≡CH]_0_ to 0.47 M (at the same [PhC≡CH]_0_/[**1**]_0_ ratio of 100), the experimental conditions approach Scenario A (the term [*S*]^*h*^ becomes smaller) and consequently, the *apparent* partial reaction order in the alkyne should increase (approaching to the maximum value of 3.62). Therefore, the value of 2.9 (entry 4, Figure 2) agrees with that expected for a system that follows eq 1.

It should be emphasized that noninteger (1.5) or excessively *high* (2.9) values, when fitting the data to the general power law *V* = *k*[*S*]^*n*^, are not directly related to the molecularity of any individual elementary step in the mechanism.

**Figure 4:**
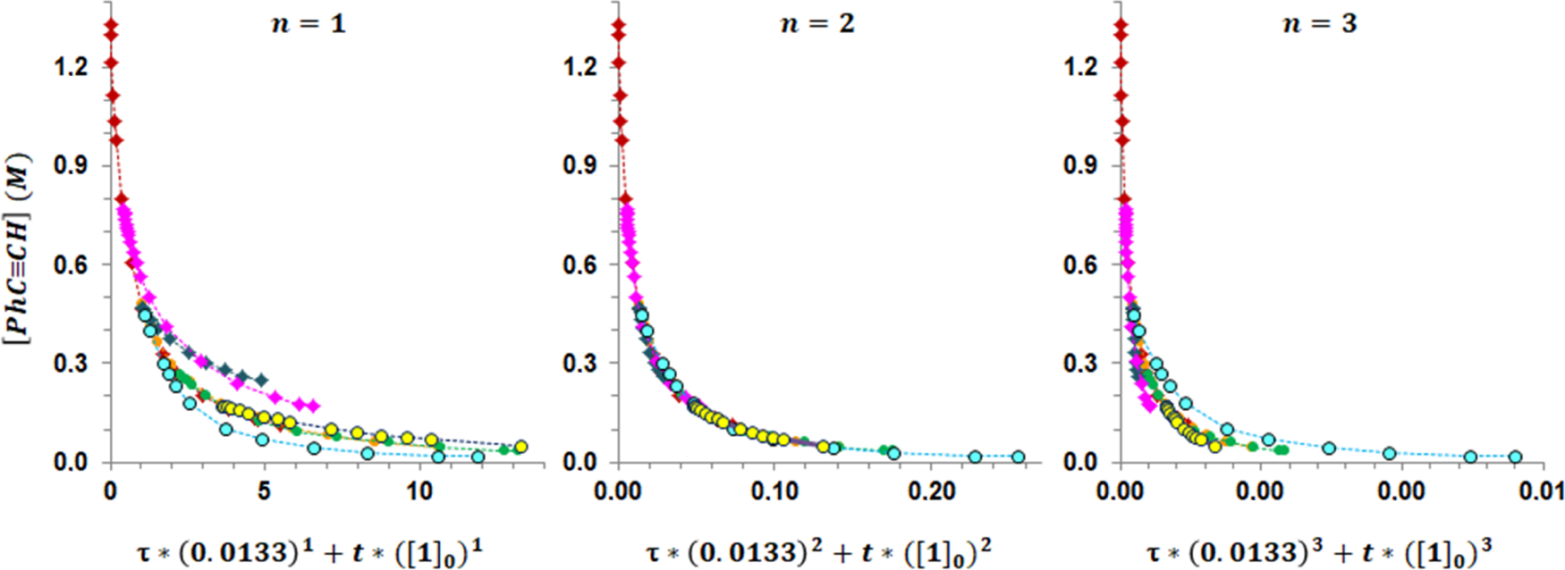
Adjusted normalized time scale plots showing the overlapping of the profiles for a partial order in the catalyst of 2. The experimental conditions can be found in Table S2: green circle (run 1), red diamond (run 2), orange diamond (run 3), blue diamond (run 4), pink diamond (run 5), light blue circle (run 6), and yellow circle (run 7). [**1**]_0_ = 0.0133 M corresponds to the reference trace (run 2).

Further insight came from the determination of the reaction order for precatalyst **1**. For these studies, the standard ‘*normalized time scale method*‘^25^ cannot be used since it requires varying [**1**]_0_ at a fixed [PhC≡CH]_0_. This methodology is associated with a change in the [PhC≡CH]_0_/[**1**]_0_ ratio, which in our case is associated with a change in the kinetics. Consequently, experiments were performed varying [**1**]_0_ at fixed [PhC≡CH]_0_/[**1**]_0_ ratios of 20 and 100 (with an additional experiment at a ratio = 36 to ensure the goodness of the results) and analyzed by the “*adjusted normalized time scale method*.”^25^ To this end, the *x*-axis of the standard [substrate] *vs* t[cat]*^n^* plots was shifted the standard time adjustment (τ) multiplied by 0.0133 (the initial concentration of **1** in the experiment that contains the highest initial concentration of phenylacetylene) to the power of the order in the catalyst. A good overlap of the substrate profiles was observed for a partial reaction order of 2 for the catalyst (Figure 4).

In addition, a plot of ln(*k*_obs_) *vs* ln([**1**]_0_) for the catalytic runs in the *low* regime (Table S2), which all displayed the same *apparent* order in [PhC≡CH] (*n* = 1.5), also indicated a partial reaction order in the catalyst of 2. A straight line with a slope of 2.08 ± 0.00011 was obtained (Figure S4). Moreover, the y-intercept gives a value for *k*_cat_ (*low*) = 35.5, which leads to *V*_max_ = *k*_cat_ ([**1**]_0_)^2^ = 0.0063 ± 0.00019 M min^–1^ for a [**1**]_0_ = 0.0133 M. This value produces a more precise sigmoidal curve for the *low* regime (Figure 3d). Conversely, the value of *V*_max_ = 0.04 M min^–1^ (Figure 3c) corresponds to a value for *k*_cat_ (*high*) = 226.1. These results allow us to conclude that the catalytically active species in the *high* regime reacts around 6 times faster than those in the *low* regime. Moreover, the homotropic cooperativity, involving up to four molecules of alkyne, accounts for the considerable 54% decrease of the reaction time (from 13 to 6 h) on increasing [PhC≡CH]_0_ from 0.26 to 1.33 M (Figure 2).

**Figure 5:**
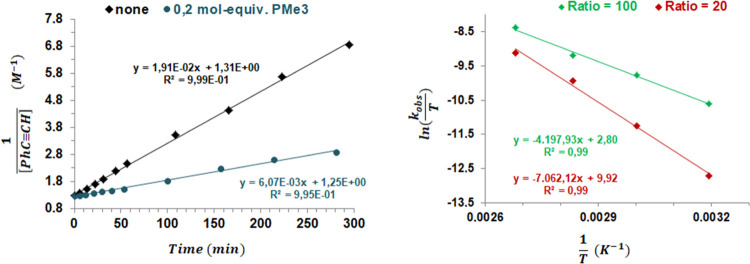
Left: Plot of 1/[PhC≡CH] (M^–1^) *vs* time (min) in the absence of additives (black diamonds) and with 0.2 mol-equiv of PMe_3_ relative to **1** (blue circles). Right: Eyring plots for the *high* (green) and *low* (red) regimes over the temperature range of 40–100 °C.

Further support for the binuclear nature of the active species was established by an additional experiment using a substoichiometric amount (0.2 mol-equiv relative to **1**) of PMe_3_ as a catalyst poison. The reaction is slower in the presence of PMe_3_ with a *k*_obs_ (*PMe*_3_)/*k*_obs_ (*none*) ratio (6.073 × 10^–3^/1.91 × 10^–2^) of 0.32 (Figure 5, left). This value fits with that expected for a second-order reaction in **1** and PMe_3_ sequestering a binuclear entity. In this scenario, PMe_3_ causes a reduction of 0.4 mol-equiv in the catalyst, leading to a ratio [**1**-PMe_3_]_0_/[**1**-*none*]_0_ of 0.6. Since *k*_*obs*_ is a function of ([**1**]_0_)^2^, a value for *k*_obs_ (*PMe*_3_)/*k*_obs_ (*none*) of 0.36 (0.6^2^) is expected.^26^

The effect of temperature was analyzed by performing the catalysis at 40, 80, and 100 °C for both regimes. At room temperature (≈25 °C), the catalysis was found to be very slow achieving only a 32% conversion after 16.5 h (*high* regime). At all temperatures studied, an *apparent* reaction order in phenylacetylene of 2 was observed for a ratio of [PhC≡CH]_0_/[**1**]_0_ = 100, but of 1.5 with a ratio of [PhC≡CH]_0_/[**1**]_0_ = 20 (Figure S5). Consequently, temperature does not affect the *apparent* reaction order in PhC≡CH, which is mainly controlled by the [PhC≡CH]_0_/[**1**]_0_ ratio.

From these data, Eyring plots of ln(*k*_obs_/*T*) *vs* 1/*T* over the temperature range of 40–100 °C give the activation parameters Δ*H*^≠^ = 8.31 kcal mol^–1^, Δ*S*^≠^ = −41.50 cal mol^–1^ K^–1^ and Δ*H*^≠^ = 13.98 kcal mol^–1^, Δ*S*^≠^ = −27.40 cal mol^–1^ K^–1^ for the *high* and *low* regimes, respectively, confirming the presence of two different catalytic cycles (Figure 5, right). The large and negative activation entropy in the *high* regime can be seen as diagnostic of a highly ordered transition state in the rate-determining step, which is less ordered in the *low* regime cycle. Conversely, this step is more endergonic in the *low* regime than in the *high* regime.

Additionally, the progress of the catalysis in the presence of equimolar amounts of PhC≡CH and PhC≡CD (ratio **1**:PhC≡CH:PhC≡CD = 1:50:50 and 1:10:10, for the *high* and *low* regimes, respectively) intermolecular competition^27^ was monitored to determine the isotope effect. This method was chosen mainly because cyclotrimerization of PhC≡CH and PhC≡CD occurs under exactly the same conditions, avoiding experimental errors, and thus the ratio of the products (*P*_H_/*P*_D_) can be measured by NMR spectroscopy with good precision. Small values of *P*_H_/*P*_D_ of 1.07 and 1.09, for the *high* and *low* regimes, respectively, were observed. Therefore, neither the rate-determining step nor the preequilibria detected by the sigmoidal behavior (Figure 2) involve the cleavage of the C–H bond.

To help identify the species formed prior to the rate-determining step, stoichiometric reactions with two and four equivalents of PhC≡CH (relative to **1**) were followed by ^1^H NMR spectroscopy. In both cases, the slow transformation of PhC≡CH into the corresponding trimers takes place, whereas complex **1** was the only observed organometallic species, a clear indication that *K*_1_ in our case is small.

**Scheme 2:**
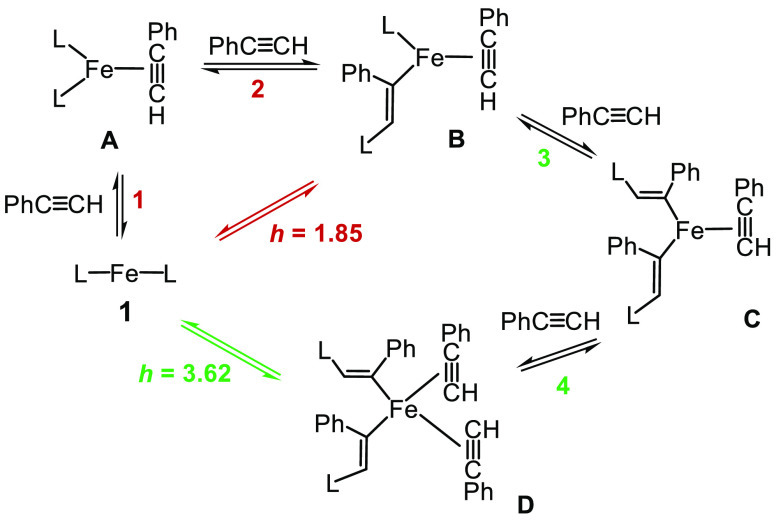
Proposed Preequilibria Undergone by Complex 1 and PhC≡CH; *L* = 2,6-Xyl_2_C_6_H_3_

**Scheme 3:**
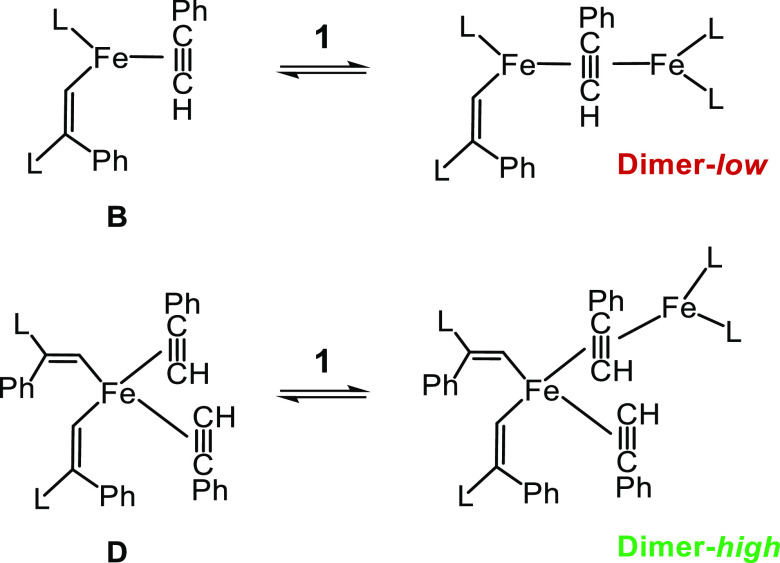
Proposed Catalytically Active Binuclear Species in the *Low* and *High* Regimes; *L* = 2,6-Xyl_2_C_6_H_3_

With the information gathered from the Eyring analysis, KIE measurements, and kinetic and stoichiometric studies, a precise proposal on what is happening in the reaction media is, unfortunately, unavailable. Nonetheless, a reasonable picture is shown in Schemes 2 and 3.

From the literature, reversible acetylene insertion has been shown to be possible using various metals (Nb,^28^ Rh,^29^ and Ru).^30^ These reactions serve as a basis for the reactivity observed as there can be no cleavage of the acetylene C–H bond during the reaction. Inserting the terphenyl into the acetylene can open up the coordination sphere, allowing more acetylene to bind. Thus, starting from complex **1**, coordination of the first molecule of PhC≡CH would give the tricoordinated species **A**.^31^ At this point, molecular models revealed that there is not enough space in **A** to accommodate a second molecule of phenylacetylene. Consequently, insertion of the acetylene into Fe–C bond is proposed to provide access to the second molecule of PhC≡CH. This insertion reaction is associated with a small change in the C–H bond (change of hybridization of the carbon from sp to sp^2^) and therefore a small KIE is expected as observed. Through this second equilibrium, species **B** would be formed. Since **B** is less crowded than **A**, equilibrium 2 is expected to be shifted to the right (*K*_2_ ≫ *K*_1_).

At a high concentration of phenylacetylene, a third molecule of PhC≡CH could reproduce the reaction to give intermediate **C** (less crowded than **B**), allowing then the easy entry of the fourth acetylene to yield **D**. The values of the Hill indexes of 1.85 and 3.62 agree with this proposal, with mainly complex **1** and intermediate **B** in the *low* regime, but **1** and **D** in the *high* regime. In this scenario, a low value for *K*_1_ is required to account for complex **1** as the only observable species even with a ratio [PhC≡CH]_0_/[**1**]_0_ = 150, the highest experimentally feasible.

The next point concerns the catalytically active species in the *low* and *high* catalytic cycles, which are both binuclear according to the second partial reaction order in complex **1**. Among the wide range of possibilities to form binuclear complexes from **B** and **D**, a reasonable proposal involves further interaction of both with complex **1**, which could give the dimer-*low* and dimer-*high* depicted in Scheme 3. The more crowded structure of the latter would account for the higher negative value of entropy estimated at the *high* regime.

In general terms, mononuclear catalysts have been mainly reported for alkyne trimerizations,^32^ a picture also found in mononuclear iron complexes and iron(0) nanoparticles. However, a novel binuclear alternative as proposed herein has been recently reported for iron cyclotrimerization,^8a^ as well as in related metal complexes.^33^

**Figure 6:**
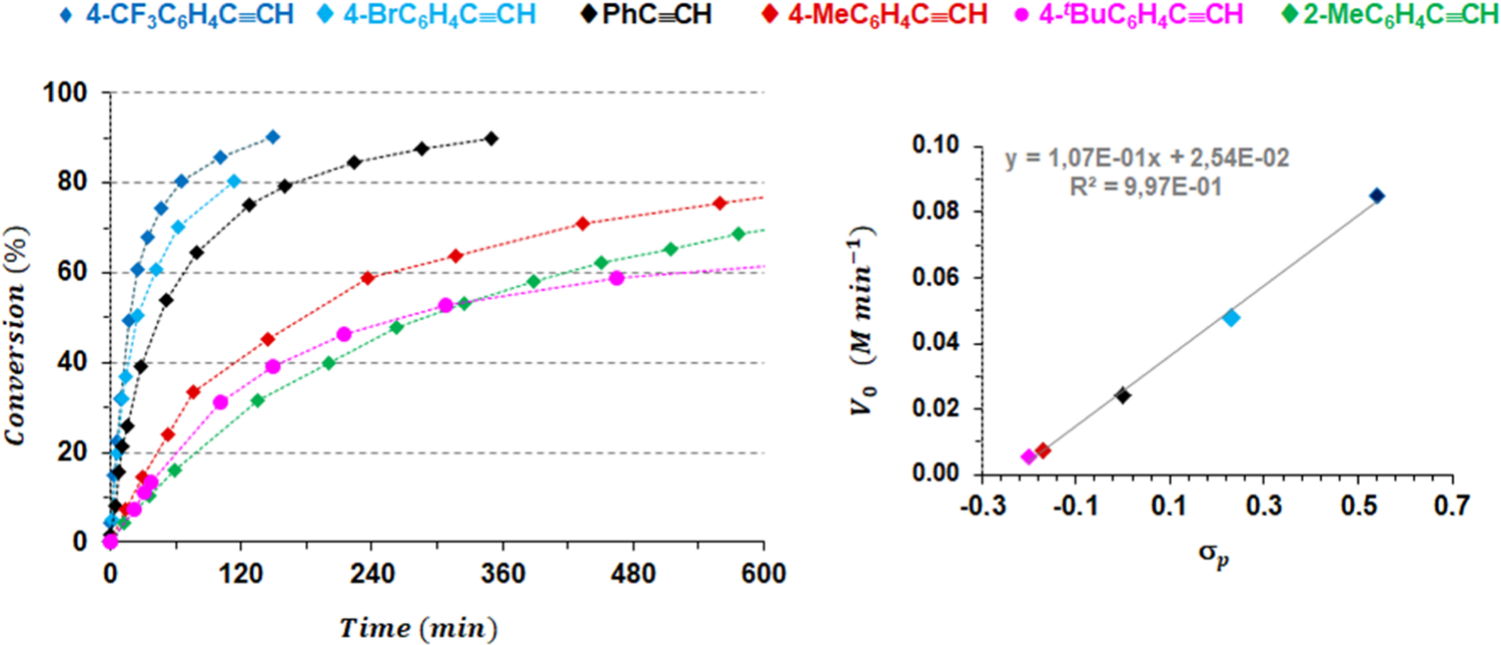
Left: Conversion (%) *vs* time (min) plot for acetylene cyclotrimerization catalyzed by **1**. Experimental conditions: [**1**]_0_ = 0.0130 M, [alkyne]_0_ = 1.33 M, *T* = 60 °C, solvent = C_6_D_6_. Dashed lines are for visual aid. Right: Plot of *V*_0_ obtained for the different alkynes *vs* their corresponding Hammett constants (σ_*p*_).

The scope of the reaction was extended to other terminal aryl acetylenes. In all cases, the catalysis is regioselective to the corresponding unsymmetrical 1,2,4-cyclotrimers (85–97%) (Table S3). In addition, Figure 6 (left) evidences the strong influence of the electronic nature of the substituent in the phenyl group on the catalysis. Thus, reducing the electronic density on the arene ring (4-CF_3_C_6_H_4_C≡CH and 4-BrC_6_H_4_C≡CH) increases the reaction rate, whereas the opposite trend was observed with the electronically richer alkynes 4-MeC_6_H_4_C≡CH and 4-^*t*^BuC_6_H_4_C≡CH. In the latter case (in pink), the catalysis slows down after 45% conversion, most likely due to an unknown impurity that deactivates the catalyst.

Moreover, plotting a graph of the initial rates (*V*_0_) *vs* Hammett constants (σ_*p*_)^34^ reveals a linear correlation (Figure 6, right), indicating a direct influence of the electronic density on the arene ring on the initial rates.

The reaction with 4-MeC_6_H_4_C≡CH (in red) is noted to be slightly faster than with the more sterically hindered isomer, 2-MeC_6_H_4_C≡CH (in green, Figure 6). This indicates that steric factors play a role in the rate of catalysis, albeit a minor one in this case.

In addition, the highly electron-deficient methyl propiolate (MeO_2_CC≡CH) immediately reacts with **1** at room temperature to afford a gummy precipitate, insoluble in common organic solvents, which is presumably polymeric. Internal alkynes such as PhC≡CPh did not undergo cyclotrimerization at 60 °C, probably due to steric effects.

As described above for phenylacetylene, the alkynes shown in Figure 6 also displayed homotropic cooperativity. This is evidenced by the *apparent* reaction order for the alkyne of 1.5 in catalytic mixtures with an [alkyne]_0_/[**1**]_0_ ratio of 20, but an *apparent* reaction order of 2 for an [alkyne]_0_/[**1**]_0_ ratio of 100 ([**1**]_0_ = 0.0133 M; Figures S6–S11).

In summary, this work demonstrates an unprecedented case of homotropic cooperativity of alkynes in regioselective [2+2+2]-cycloadditions to 1,2,4-(aryl)-benzenes. The kinetic behavior is strongly dependent on the [RC≡CH]_0_/[**1**]_0_ ratio in such a way that two alkyne molecules cooperate at ratios <40, whereas up to four molecules cooperate at ratios >40. Values for the Hill index of 1.85 and 3.62 strongly support a noteworthy high level of positive substrate cooperation in both scenarios. Moreover, active species in the *high* regime were found to react *ca.* 6 times faster than those involved in the *low* regime. Accordingly, two sets of parameters for the *high* and *low* regimes, respectively, confirming the presence of two different catalytic cycles, were obtained from Eyring analyses. The rate-determining step in the *low* regime was found to be more endergonic than in the *high* one, with a more ordered transition state in the *high* region than in the *low* one. We believe that our findings reveal unique features of the kinetic behavior of alkynes, providing key insights into detecting and analyzing these atypical phenomena in reactions catalyzed by organometallic complexes.

## Supporting Information Available

General information, experimental procedures, kinetic studies, and NMR spectra of isolated tri-(aryl)benzenes (PDF)
