# Supplementary material for: Homotropic Cooperativity in Iron-Catalyzed Alkyne Cyclotrimerizations
Source: ACS Catal. 2023 Apr 28;13(10):6610–8. doi: 10.1021/acscatal.3c00764 (PMC10204060; doi:10.1021/acscatal.3c00764)
Supplement: Supplementary file 1 — cs3c00764_si_001.pdf [file cs3c00764_si_001.pdf]

Homotropic Cooperativity in Iron-Catalyzed Alkyne Cyclotrimerizations

Ana M. Geer,[a,*] Janeth Navarro,[a] Pablo Alamán-Valtierra,[a] Nathan T. Coles,[b] Deborah L. Kays,[b,*] and Cristina Tejel[a,*]

[a] Instituto de Síntesis Química y Catálisis Homogénea (ISQCH), departamento de química inorgánica, CSIC-Universidad de Zaragoza, Pedro Cerbuna 12, Facultad de Ciencias, 50009-Zaragoza (Spain), email: ctejel@unizar.es, anageer@unizar.es

[b] School of Chemistry, University of Nottingham, University Park, Nottingham, NG7 2RD, U.K, email: Deborah.Kays@nottingham.ac.uk

Experimental Procedures.

Starting materials and physical methods.

All manipulations were carried out under strictly anhydrous and anaerobic conditions using glovebox facilities. Complex [$Fe(2,6\text{-}Xyl_2C_6H_3)_2$] (**1**, Xyl = 2,6-$Me_2C_6H_3$) was prepared according to literature methods.[S1] Commercially available alkynes were distilled under reduced pressure prior to use. C_6D_6 was dried over a liquid Na/K alloy for a week. A solution of PMe_3 (1 M in toluene) was purchased from Sigma-Aldrich and used directly.

NMR spectra were recorded on Bruker AV300, AV400 and AV500 spectrometers operating at 300.13, 400.13, and 500.13 MHz, respectively, for ^1H. Chemical shifts are reported in ppm and referenced to $SiMe_4$, using the internal signal of the deuterated solvent (^1H and ^{13}C) and external CF_3COOH (^{19}F, −76.6 ppm *vs* $CFCl_3$).

^1H NMR spectra for quantitative measurements were recorded using the standard sequence from Bruker zg30, with 16 scans, a delay (d1) of 2 s, and an adquisition time (aq) of 3 s, which ensures a good relative integral of the selected resonances of complexes/substrates/products and that from the internal standard (the CH_2 singlet of dioxane). In selected cases, experiments were carried out using $^tBuC_6H_5$ as internal standard (whithout dioxane). Similar profiles were observed, which ensures that dioxane does not influence the data.

For temperature calibration a reference tube with 100% ethylene glycol was used, where the chemical-shift separation (Δ) in ppm between the CH_2 and the OH peaks, is related to the temperature by the formula $T = (4.637-\Delta)/0.009967$.

Kinetic studies

For all studies, a stock solution in C_6D_6 containing complex **1** (30.0 mg, 0.048 mmol) and dioxane 37 μL (0.433 mmol) was prepared in a 2 mL volumetric flask in the glovebox. The stability of **1** in solution was checked by ^{1}H NMR spectroscopy for several days. No changes in the integral of selected resonances of **1** relative to the singlet of dioxane were apparent during the first two days at room temperature, whereas the concentration of **1** started to slightly diminish in the third day. Therefore, the stock solution was used within two days.

Initial rates at different phenylacetylene loadings and monitoring of the reactions

For estimation of the initial rate (V_0), 278 μL of the stock solution were transferred to a J-Young NMR tube. Then, the appropriate amount of C_6D_6 (to get a total volume of 500 μL) and different loadings of phenylacetylene were carefully added. Through this procedure a $[\mathbf{1}]_0$ = 0.0133 M is present in all the tubes. The NMR tube was sealed, removed from the glovebox, manually shaken in the NMR room for 15 s, and introduced into the NMR probe preheated to 60 ºC. The reactions were monitored by ^{1}H NMR spectroscopy, recording ^{1}H NMR spectra at intervals of 3-5 min, up to conversions of *ca.* 30-40%. The time at which each spectrum was acquired was directly taken from the Mestre-software (view/table/parameters). Initial rates were then measured from the linear regression fit of [PhC≡CH] versus time for the first data that gave a good straight line (generally up to a 10-12% conversion) (Table S1).

Table S1. Initial rates (V_0) for the cyclotrimerization of phenylacethylene catalyzed by **1** (0.0133 M) at different PhC≡CH loadings in C_6D_6 at 60 ºC.

[PhC≡CH]$_0$ (M)	V_0 (M min^{-1})	[PhC≡CH]$_0$/[**1**]$_0$	$1/V_0$	1/[PhC≡CH]$_0$
1.830	$3.310•10^{-2}$	**137.6**	30.21	0.55
1.452	$2.666•10^{-2}$	**109.2**	37.51	0.69
1.330	$2.423•10^{-2}$	**100.0**	41.27	0.75
1.328	$2.428•10^{-2}$	**99.8**	41.19	0.75
1.196	$2.048•10^{-2}$	**89.9**	48.83	0.84
1.180	$1.949•10^{-2}$	**88.7**	51.31	0.85
1.063	$1.579•10^{-2}$	**77.0**	63.33	0.94
0.914	$1.080•10^{-2}$	**68.7**	92.59	1.09
0.675	$4.704•10^{-3}$	**50.8**	212.59	1.48
0.479	$2.260•10^{-3}$	**35.9**	442.48	2.09
0.309	$1.185•10^{-3}$	**23.3**	843.88	3.23
0.266	$1.018•10^{-3}$	**20.0**	982.32	3.76
0.219	$7.462•10^{-4}$	**16.4**	1340.12	4.57
0.141	$3.190•10^{-4}$	**10.6**	3134.80	7.09
0.0664	$9.100•10^{-5}$	**5.0**	10989.01	15.06

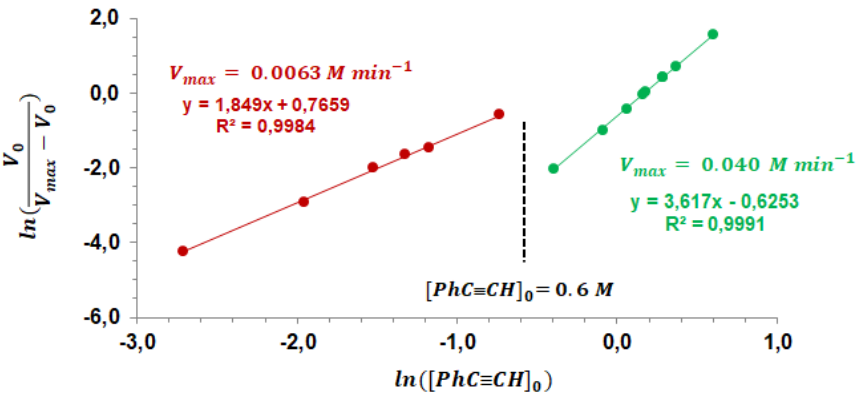

Figure S1. Linear plot of the Hill equation for the *low* (left, in red) and *high* (right, in green) regimes, each one with its corresponding V_{max}.

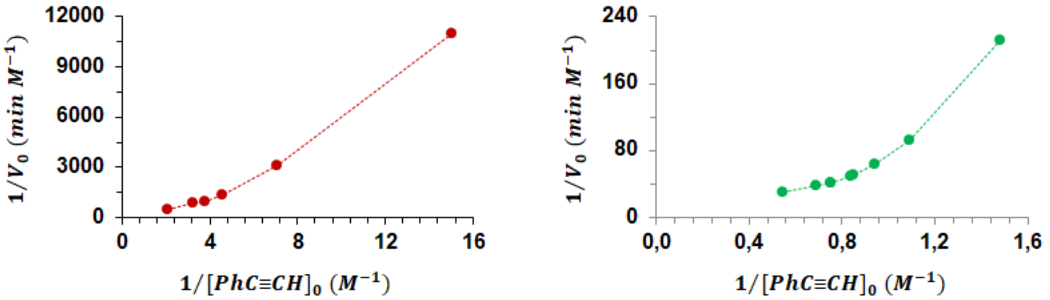

Figure S2. Lineweaver-Burk replots for the *low* (left) and *high* (right) regimes. Dashed lines are for visual aid. An upward curve indicating positive cooperativity is observed. A linear plot indicates no-cooperativity, whereas a downward curve corresponds to negative cooperativity.

Partial reaction order of the catalyst (1)

Samples for these studies were prepared in J-Young NMR tubes as described above and the reactions were monitored by ^{1}H NMR spectroscopy for 12-14 h. A representative range of experiments with [**1**]$_0$ (from 0.0224 to 0.00470 M) and [PhC≡CH]$_0$/[**1**]$_0$ (20, 36 and 100) were analyzed (Table S2).

Table S2. Experimental conditions for the determination of the partial reaction order in **1**.[a]

Entry	[**1**]$_0$ (M)	Mol-cat. (%)	[PhC≡CH]$_0$ (M)	[PhC≡CH]$_0$/[**1**]$_0$	n[b]	Time adjustment (τ) (min)	k_{obs}
1	0.0133	5	0.266	20	**1.5**	168	$4.35 \cdot 10^{-3}$
2	0.0133	1	1.330	100	2	0	
3	0.0133	3	0.479	36	**1.5**	77	$4.47 \cdot 10^{-3}$
4	0.00470	1	0.470	100	2.9	78	
5	0.00771	1	0.771	100	2.1	32	
6	0.0224	5	0.449	20	**1.5**	83	$1.31 \cdot 10^{-2}$
7	0.00856	5	0.171	20	**1.5**	272	$1.77 \cdot 10^{-3}$
8	0.0062	5	0.124	20	**1.5**	370	$9.00 \cdot 10^{-4}$

[a] T = 60 ºC, in C_6D_6. [b] *Apparent* partial reaction order in PhC≡CH

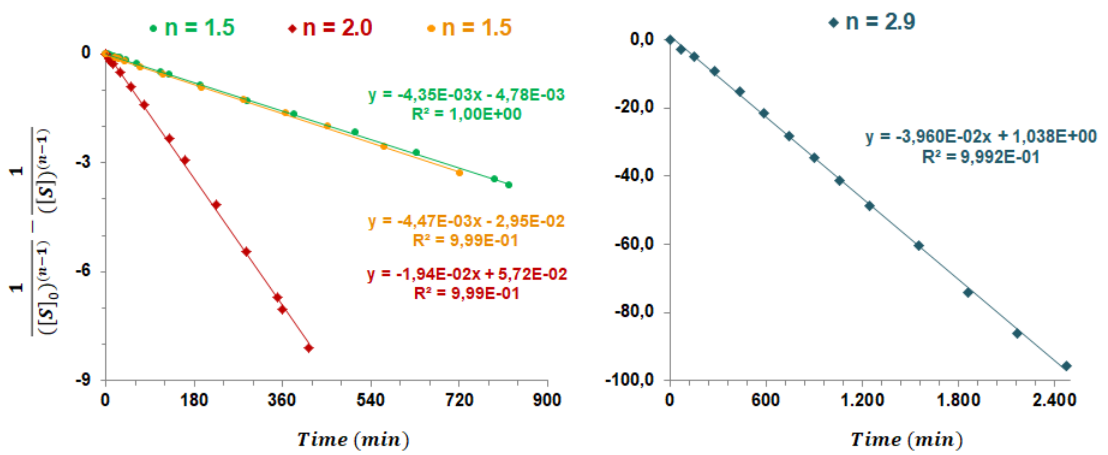

Figure S3. Kinetic profiles for reactions in Figure 2 in the main manuscript. S = PhC≡CH. Code: green circle (run 1), orange circle (run 3), red diamond (run 2), blue diamond (run 4), in Table S2 and Figure 2 in the main manuscript.

Additionally, for runs 1, 3, 6, 7, and 8, which showed comparable kinetic profiles (n = 1.5), a plot of $ln(k_{obs})\ vs\ ln([\mathbf{1}]_0)$ gave a straight line with a slope of 2, again in agreement with a partial reaction order of 2 in complex **1** (Figure S4). The y-intercept (3.57±0.057) corresponding to $ln(k_{cat})$ provides a more precise value of 0.0063±0.00019 M min^{-1} for $V_{max} = k_{cat}([\mathbf{1}]_0)^2$ for the *low* regime at $[\mathbf{1}]_0$ = 0.0133 M.

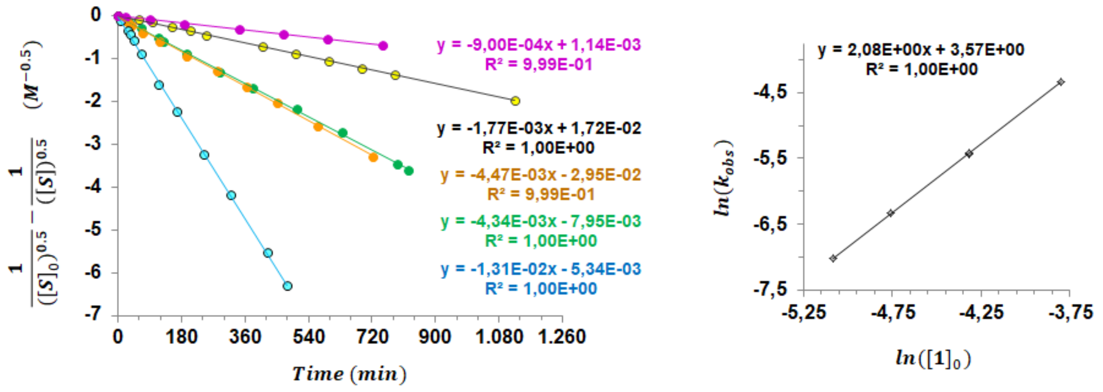

Figure S4. Kinetic profiles for reactions showing an *apparent* reaction order in phenylacetylene of 1.5 (left) and $\ln(k_{obs})$ *vs* $\ln([\mathbf{1}]_0)$ plot (right). S = PhC≡CH. Color code: green (run 1), orange (run 3), light blue (run 6), yellow (run 7), and purple (run 8) in Table S2.

Eyring plots

Solutions for the *high* regime ([**1**]$_0$ = 0.0133 M and [PhC≡CH]$_0$ = 1.33 M) were prepared transferring 278 µL of the stock solution (similar to that above described) to a J-Young NMR tube, and adding C_6D_6 (149 µL) and phenylacetylene (73 µL) to get a total volume of 500 µL. The NMR tubes were sealed, removed from the glovebox, manually shaken in the NMR room for 15 s, and introduced into the NMR probe preheated to 40, 80 and 100 ºC. An *apparent* reaction order in phenylacetylene of 2 was observed for all studied temperatures (Figure S5, left). Samples for the analysis in the *low* regime ([**1**]$_0$ = 0.0133 M and [PhC≡CH]$_0$ = 0.266 M) were prepared in a similar way but adding 15 µL of phenylacetylene and 207 µL C_6D_6. An *apparent* reaction order in phenylacetylene of 1.5 was observed for all studied temperatures (Figure S5, right). From this data, two independent Eyring plots for the *high* and *low* regimes were obtained. They are shown in Figure 5 (right) in the main text.

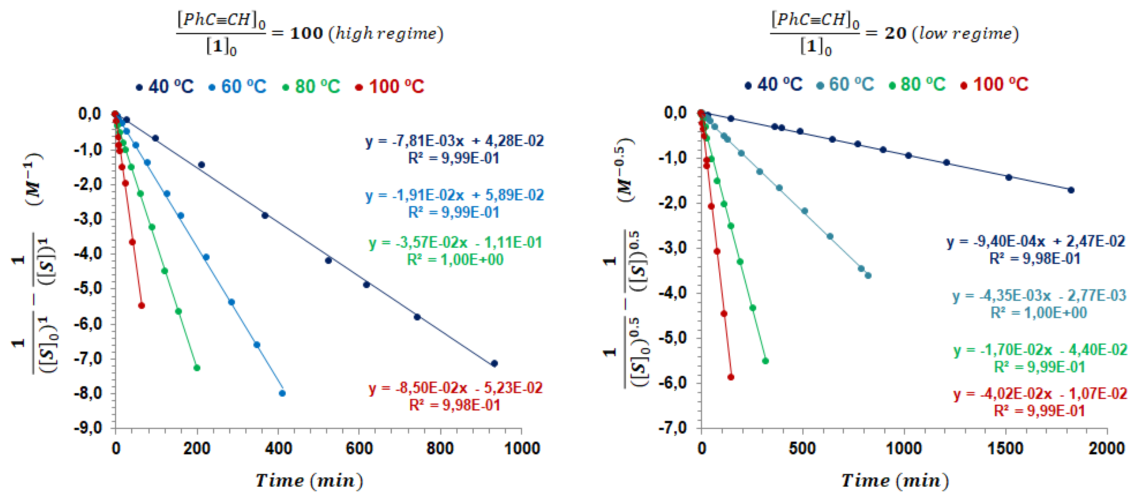

Figure S5. Kinetic profiles for reactions at 40, 60, 80 and 100 ºC for the *high* (left) and *low* (right) regimes.

Catalysis in the presence of PMe_3 and Kinetic Isotope Effects

A J-Young NMR tube was prepared in the glove-box using 278 μL of a similar stock solution as above described. Then, C_6D_6 (149 μL) and freshly distilled phenylacetylene (73 μL, 0.665 mmol) were carefully added. The NMR tube was sealed, removed from the glovebox, manually shaken in the NMR room for 15 s and immediately loaded into the NMR spectrometer preheated at 60 ºC. The reaction was monitored by ^1H NMR spectroscopy for 5 h (black diamonds, Figure 5 in the main text). After that, a second identical J-Young NMR tube was prepared and then PMe_3 (1 M in toluene, 1.33 μL, 0.2 mol-equiv. relative to **1**) was added (red diamonds, Figure 5). The exact amount of PMe_3 in the NMR tube was verified by comparing the integral of the methyl group of toluene relative to the internal standard. Since these experiments were carried out under identical conditions for $[\mathbf{1}]_0$ and $[PhC{\equiv}CH]_0$, the slope of the profiles (k_{obs}) are comparable and they can be used to estimate the influence of the additive.

NMR tubes for kinetic isotope effect (KIE) measurements (intermolecular competition) were prepared as above from a similar stock solution. Thus, 278 μL of the stock solution, C_6D_6 (185 μL), and equimolar amounts (0.333 mmol) of PhC≡CH (36 μL) and PhC≡CD (98.6 %, 37 μL)) were added to a J-Young NMR tube for measurements in the high-regime. For those in the low-regime, the J-Young NMR tube was prepared by adding 278 μL of the stock solution, C_6D_6 (215 μL), 7.2 μL of PhC≡CH and 7.4 μL of PhC≡CD (0.067 mmol of each). When reactions reached completion the relative amounts of deuterated to non-deuterated cyclotrimers were measured by integration of the quantitative ^1H NMR spectrum. Values of P_H/P_D of 1.07 and 1.09 were obtained from the high and low regimes, respectively.

Catalytic Experiments.

In a typical experiment, the amount of **1** was weighed into a vial in the glovebox (3.8 – 4.2 mg) and with this value the amount of alkyne (100/20 mol-equiv. for the *high*/*low* regimes, respectively) and solvent (C_6D_6) were calculated to ensure that the initial concentration ($[\mathbf{1}]_0$ = 0.0133 M) were the same for all experiments. Then, C_6D_6, dioxane (5 μL, 0.058 mmol) and the alkyne were added to the vial. In the case of solid 4-$BrC_6H_4C{\equiv}CH$, the alkyne was weighed at the beginning. Immediately 450 μL of these solutions were transferred to a J-Young NMR tube. The NMR tube was sealed, removed from the glovebox, and loaded into the NMR spectrometer preheated at 60 ºC (around 3-7 min). ^1H NMR spectra were recorded at different intervals of time. All reactions were monitored by NMR spectroscopy up to conversions of 60-90%. Conversions were calculated from the integration of the alkyne proton and selectivities from the integration of selected protons of the cyclotrimers relative to the internal standard (Table S3). For 4-$CF_3C_6H_4C{\equiv}CH$, the **2**:**3** ratio was estimated from the $^{19}F\{^1H\}$ NMR spectra. An additional experiment (carried out under similar conditions) with the internal alkyne $PhC{\equiv}CPh$ revealed no conversion after 66 h.

Table S3. Cyclotrimerization of alkynes catalyzed by complex **1**.

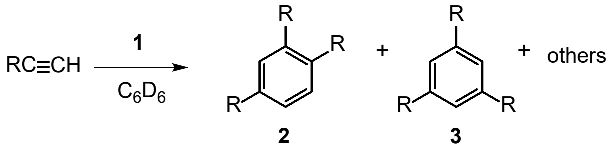

Alkyne	T (ºC)	Conv. (%)	Time (min)	**2**:**3**:others	**2**:**3**
**$[1]_0$ = 0.0133 M $[RC{\equiv}CH]_0/[1]_0$ = 100**					
4-$CF_3C_6H_4C{\equiv}CH$	60	90	150	90:3:7	97:3
4-$BrC_6H_4C{\equiv}CH$	60	81	113	62:5:33	93:7
PhC≡CH	60	90	350	80:13:7	86:14
4-$MeC_6H_4C{\equiv}CH$	60	79	685	69:12:19	85:15
4-$^tBuC_6H_4C{\equiv}CH$	60	68	1380	59:8:33	88:12
2-$MeC_6H_4C{\equiv}CH$	60	75	800	83:9:8	90:10
PhC≡CPh	60	0	3900	----	----
**$[1]_0$ = 0.0133 M $[RC{\equiv}CH]_0/[1]_0$ = 20**					
4-$CF_3C_6H_4C{\equiv}CH$	60	95	253	84:8:8	91:9
4-$BrC_6H_4C{\equiv}CH$	60	83	255	57:13:30	82:18
PhC≡CH	60	87	790	76:11:13	87:13
4-$MeC_6H_4C{\equiv}CH$	60	81	826	58:14:28	81:19
4-$^tBuC_6H_4C{\equiv}CH$	60	71	706	74:14:11	84:16
2-$MeC_6H_4C{\equiv}CH$	60	64	760	74:12:14	86:14

Fitting the data to the general equation for a nth-order reaction for the substrate:

$$\frac{1}{([S])^{(n-1)}} = k(n-1)t + \frac{1}{([S]_0)^{(n-1)}}$$

that corresponds to the integrated rate law for the power law: $rate = k[S]^n$, was used to ensure that all the tested substrates showed a similar kinetic response: an *apparent* partial reaction order of 2 in the alkyne if $[PhC{\equiv}CH]_0/[\mathbf{1}]_0 \approx 100$, but of 1.5 if $[PhC{\equiv}CH]_0/[\mathbf{1}]_0 \approx 20$ for $[\mathbf{1}]_0 = 0.0133$

M (Figures S6-S11). As commented in the manuscript, these values are not directly related to the molecularity of any individual elementary step in the mechanism.

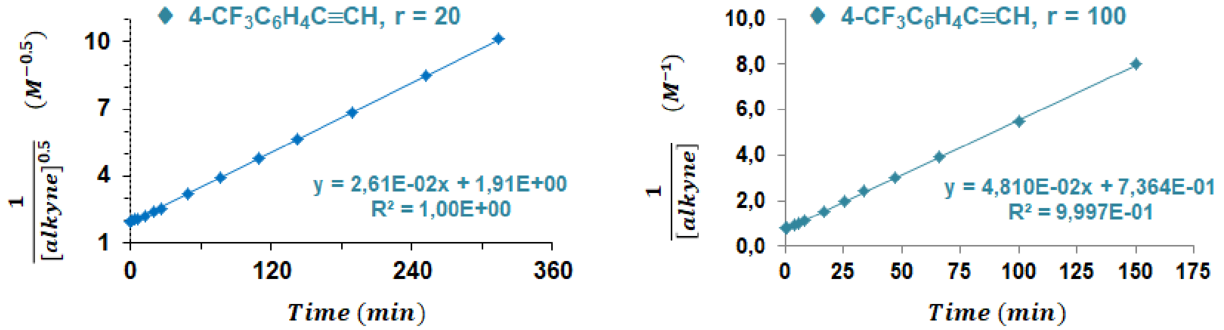

Figure S6. Plots of 1/[alkyne]$^{0.5}$ ($M^{-0.5}$) (left) and 1/[alkyne] (M^{-1}) (right) and *vs* time (min) for the catalytic cyclotrimerization of 4-$CF_3C_6H_4C$≡CH at 60 ºC in C_6D_6 and with [alkyne]$_0$/[**1**]$_0$ ratios (r) of 20 (left) and 100 (right).

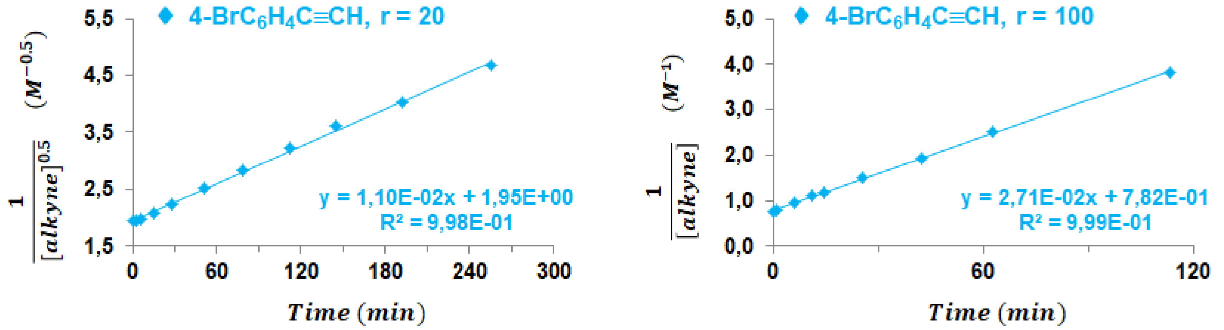

Figure S7. Plots of 1/[alkyne]$^{0.5}$ ($M^{-0.5}$) (left) and 1/[alkyne] (M^{-1}) (right) and *vs* time (min) for the catalytic cyclotrimerization of 4-BrC_6H_4C≡CH at 60 ºC in C_6D_6 and with [alkyne]$_0$/[**1**]$_0$ ratios (r) of 20 (left) and 100 (right).

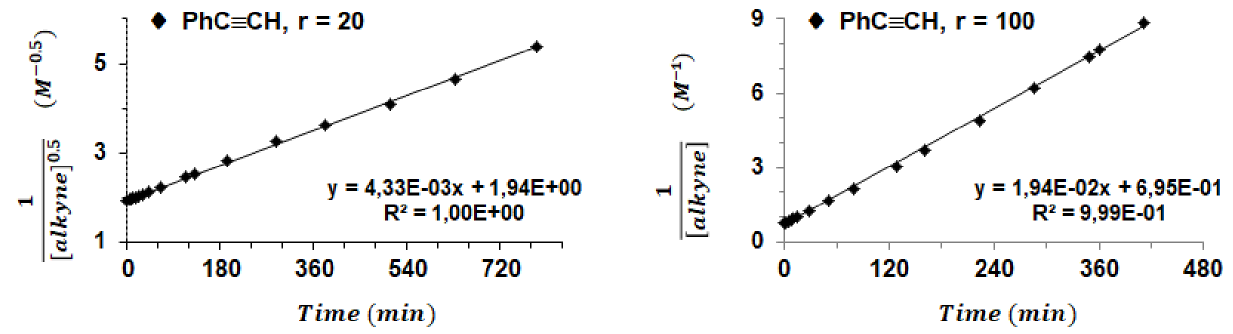

Figure S8. Plots of 1/[alkyne]$^{0.5}$ ($M^{-0.5}$) (left) and 1/[alkyne] (M^{-1}) (right) and *vs* time (min) for the catalytic cyclotrimerization of PhC≡CH at 60 ºC in C_6D_6 and with $[alkyne]_0/[\mathbf{1}]_0$ ratios (r) of 20 (left) and 100 (right).

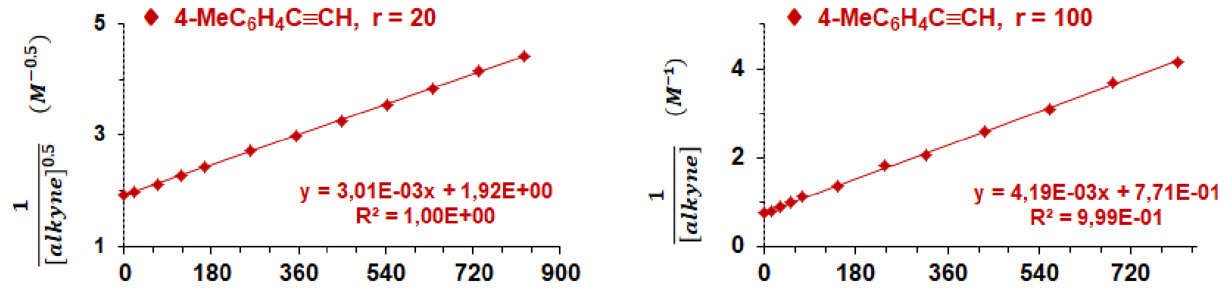

Figure S9. Plots of 1/[alkyne]$^{0.5}$ ($M^{-0.5}$) (left) and 1/[alkyne] (M^{-1}) (right) and *vs* time (min) for the catalytic cyclotrimerization of 4-MeC_6H_4C≡CH at 60 ºC in C_6D_6 and with $[alkyne]_0/[\mathbf{1}]_0$ ratios (r) of 20 (left) and 100 (right).

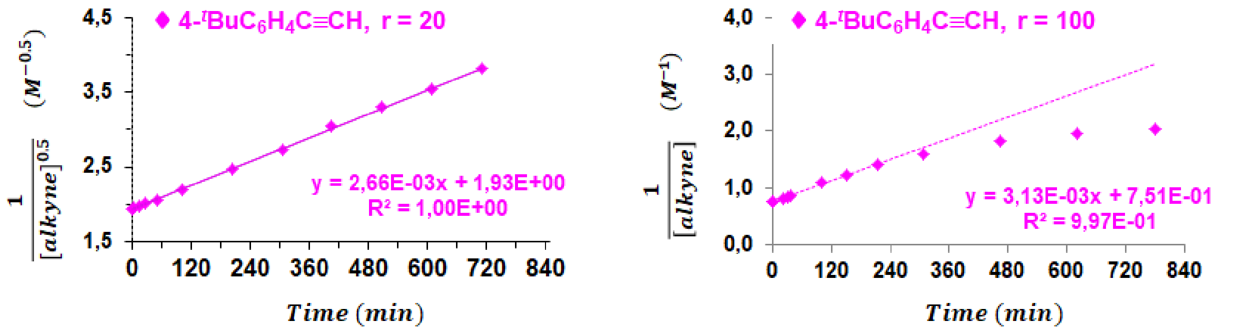

Figure S10. Plots of 1/[alkyne]$^{0.5}$ ($M^{-0.5}$) (left) and 1/[alkyne] (M^{-1}) (right) and *vs* time (min) for the catalytic cyclotrimerization of 4-$^{t}BuC_6H_4C$≡CH at 60 ºC in C_6D_6 and with $[alkyne]_0/[\mathbf{1}]_0$ ratios (r) of 20 (left) and 100 (right). In the *high* regime, the catalysis slows down after 45% of conversion, probably due to an unknown impurity that destroys the catalyst.

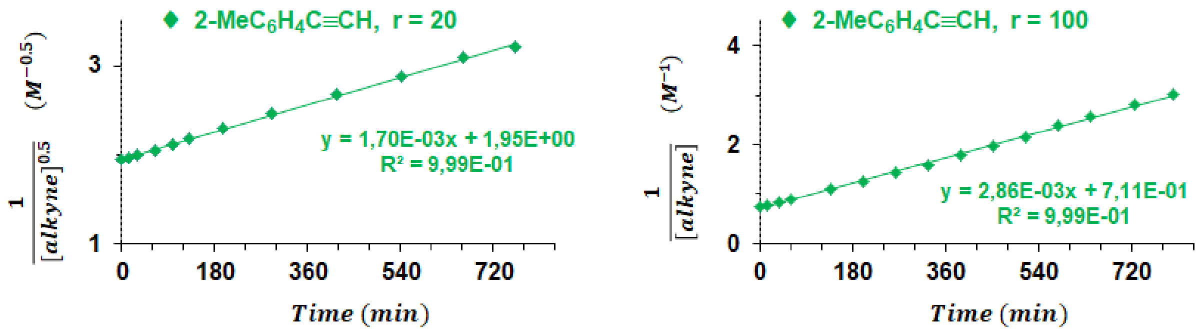

Figure S11. Plots of 1/[alkyne]$^{0.5}$ ($M^{-0.5}$) (left) and 1/[alkyne] (M^{-1}) (right) and *vs* time (min) for the catalytic cyclotrimerization of 2-$MeC_6H_4C{\equiv}CH$ at 60 ºC in C_6D_6 and with $[alkyne]_0/[\mathbf{1}]_0$ ratios (r) of 20 (left) and 100 (right).

Characterization of tri-(aryl)benzenes

Once the catalysis with an $[alkyne]_0/[\mathbf{1}]_0$ ratio of 100 reached the conversions shown in Table S2, the solutions were transfered to a Schlenk tube, dichloromethane (2 x 1 mL) was used to complete the transfer, and the solutions were evaporated to dryness. The cyclotrimers were extracted with two portions of *n*-hexane (2 mL) at 45 ºC. The suspensions were decanted and filtered over diatomaceous earth. Evaporation of the filtrates to dryness afforded the cyclotrimers, generally as oils. Both the filtrates and the residues were analyzed by NMR spectrocopy. Small amounts of cyclotrimers (less than 2%) reminained in the residue. In the particular case of tri-(4'-trifluoromethylphenyl)benzene, pure 1,2,4-isomer was found in the residue. The cyclotrimers were analyzed by multinuclear NMR spectroscopy in C_6D_6, which allowed known signals to be integrated at the end of the catalysis. Tri-(2'-methylphenyl)benzene was found to be fluxional in C_6D_6 at rt, but reasonably sharp spectra were obtained at 80 ºC. For selected NMR spectra of the cyclotrimers see Figures S12-S17.

1,2,4-Tri-(4'-trifluoromethylphenyl)benzene. Isolated yield = 63% (filtrate) + 20% (residue) = 83%.

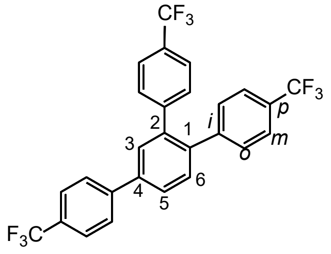

^{1}H NMR (500.13 MHz, C_6D_6, 25 ºC): δ = 7.50 (d, J = 8.1 Hz, 2H, Ar), 7.36 (d, J = 1.9 Hz, 1H, H^3), 7.33 (dd, J = 7.9, 1.9 Hz, 1H, H^5), 7.31 (d, J = 8.3 Hz, 2H, Ar), 7.212 (d, J = 7.8 Hz, 2H, Ar), 7.21 (d, J = 8.3 Hz, 2H, Ar), 7.20 (d, J = 7.7 Hz, 1H, H^6), 6.93 (d, J = 8.7 Hz, 2H, Ar), 6.91 (d, J = 8.6 Hz, 2H, Ar); 1,3,5-isomer: δ = 7.53 (d, J = 8.1 Hz, 6H, Ar), 7.49 (s, 3H, H^2), 7.31 (d, J = 8.3 Hz, 6H, Ar).

^{13}C{^{1}H} NMR (125.8 MHz, C_6D_6, 25 ºC): δ = 144.6 (q, J(C,F) = 1 Hz), 144.3 (q, J(C,F) = 1 Hz), and 143.8 (q, J(C,F) = 1 Hz) (C^i Ar), 140.2 (2C) and 139.2 (C^1, C^2, C^4), 131.7 (C^6), 130.2 (q, J(C,F) = 33 Hz), 129.7 (q, J(C,F) = 33 Hz), and 129.6 (q, J(C,F) = 33 Hz) (C^p), 130.39, 130.35, and 127.7 (C^o), 129.9 (C^3), 127.3 (C^5), 126.2 (q, J(C,F) = 3.7 Hz), 125.49 (q, J(C,F) = 4 Hz), and 125.47 (q, J(C,F) = 4 Hz) (C^m), 125.0 (q, J(C,F) = 272 Hz), 124.81 (q, J(C,F) = 272 Hz), and 124.79 (q, J(C,F) = 272 Hz) (CF_3).

^{19}F{^{1}H} NMR (470.6 MHz, C_6D_6, 25 ºC):δ = −62.16, −62.25 (2F); 1,3,5-isomer: δ = −62.14.

HR-APCI-MS (m/z): calcd for $C_{27}H_{15}F_9$ $[M]^+$ 510.1025, found 510.1011 (err [mDa] = −1.4).

1,2,4-Tri-(4'-bromomethylphenyl)benzene. Isolated yield = 67%

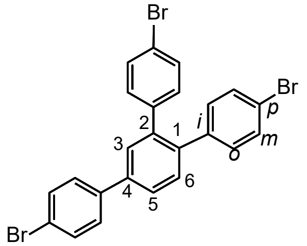

^{1}H NMR (500.13 MHz, C_6D_6, 25 ºC): δ = 7.38 (d, J = 8.5 Hz, 2H, Ar), 7.33 (d, J = 2.0 Hz, 1H, H^3), 7.28 (dd, J = 8.0, 2.0 Hz, 1H, H^5), 7.17 (d, partially overlapped by the solvent signal, 1H, H^6), 7.12 (m, 6H, Ar), 6.75 and 6.72 (both ddd, J = 8.0, 2.0 Hz, 2H each, Ar); 1,3,5-isomer: δ = 7.42 (s, 3H, H^2), 7.40 (d, J= 8.5 Hz, 6H, Ar), 7.11 (d, partially overlapped, 6H, Ar).

^{13}C{^{1}H} NMR (125.8 MHz, C_6D_6, 25 ºC): δ = 140.2, 140.1, 140.0, 139.9, 139.4, and 138.8 (C^1, C^2, C^4, C^i), 132.4, 131.72, 131.69 (3C) and 131.67 ($C^{o,m}$), 131.5 (C^6), 129.4 (C^3), 126.6 (C^5), 122.4, 121.74, and 121.71 (C^p); 1,3,5-isomer: δ = 141.8 and 136.0 (C^1, C^i), 132.3, and 129.23 ($C^{o,m}$), 125.3 (C^2), 122.4 (C^p).

HR-APCI-MS (m/z): calcd for $C_{24}H_{15}Br_3$ $[M]^+$ 539.8718, found 539.8703 (err [mDa] = −1.5).

1,2,4-Tri-(phenyl)benzene. Isolated yield: 82%.

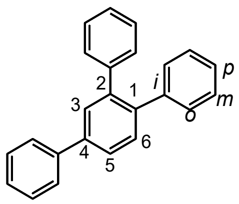

^{1}H NMR (500.13 MHz, C_6D_6, 25 ºC): δ = 7.69 (d, *J* = 1.9 Hz, 1H, H^3), 7.52 (d, *J* = 7.5 Hz, 2H, H^o), 7.50 (dd, *J* = 7.9, 1.9 Hz, 1H, H^5), 7.41 (d, *J* = 7.9 Hz, 1H, H^6), 7.27–7.15 (m, 7H, Ph), 7.09–6.98 (m, 6H, Ph);

selected resonances for the symetrical 1,3,5-isomer: δ = 7.78 (s, 1H, H^2)

^{13}C{^{1}H} NMR (125.8 MHz, C_6D_6, 25 ºC): δ = 142.2, 141.8, 141.6, 141.4, 140.9, 140.0 (C^1, C^2, C^4, C^i), 131.6 (C^6), 130.0 (C^3), 130.33, 130.29, 129.1, 128.3 (2C), 127.6, 127.1, 126.88, 126.85 ($C^{o,m,p}$), 126.5 (C^5), selected resonances for the symetrical 1,3,5-isomer: δ = 129.1, 127.8, 127.7 ($C^{o,m,p}$), 125.6 (C^2).

HR-APCI-MS (m/z): calcd for $C_{24}H_{18}$ $[M]^+$ 306.1403, found 306.1397 (err [mDa] = –0.6).

1,2,4-Tri-(4'-methylphenyl)benzene. Isolated yield = 63%

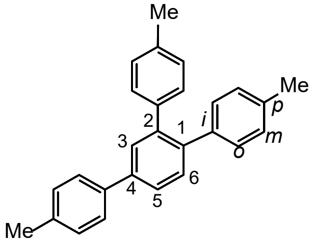

^{1}H NMR (500.13 MHz, C_6D_6, 25 ºC): δ = 7.79 (d, *J* = 2.0 Hz, 1H, H^3), 7.56 (dd, *J* = 7.9, 2.0 Hz, 1H, H^5), 7.52 (d, *J* = 8.1 Hz, 2H, Ar), 7.48 (d, *J* = 7.9 Hz, 1H, H^6), 7.21 (d, *J* = 8.0 Hz, 2H, Ar), 7.19 (d, *J* = 8.1 Hz, 2H, Ar), 7.09 (d, *J* = 7.6 Hz, 2H, Ar), 6.91 (d, *J* = 7.5 Hz, 2H, Ar), 6.90 (d, *J* = 7.6 Hz, 2H, Ar), 2.18 (s, 3H, Me), 2.06 (s, 3H, Me), 2.05 (s, 3H, Me); 1,3,5-isomer: δ = 7.87 (s, 3H, H^2), 7.54 (d, *J* = 8.1 Hz, 6H, Ar), 7.11 (d, *J* = 9.6 Hz, 6H, Ar), 2.19 (s, 9H, Me).

^{13}C{^{1}H} NMR (125.8 MHz, C_6D_6, 25 ºC): δ = 141.6, 140.7, 139.8, 139.6, 139.2, and 138.5 (C^1, C^2, C^4, C^i), 137.1, 136.2, and 136.1 (C^p), 131.6 (C^6), 129.8 (C^3), 130.3, 130.2, 129.9, 129.1 (2C), and 127.4 ($C^{o,m}$), 126.2 (C^5), 21.12, and 21.07 (2C) (Me); 1,3,5-isomer: δ = 142.9 and 139.1 (C^1, C^i), 137.2 (C^p), 129.86, and 127.7 ($C^{o,m}$), 125.1 (C^2), 21.14 (Me).

HR-APCI-MS (m/z): calcd for $C_{27}H_{24}$ $[M]^+$ 348.1873, found 348.1857 (err [mDa] = –1.6).

1,2,4-Tri-(4'-^{t}butylphenyl)benzene. Isolated yield = 67%

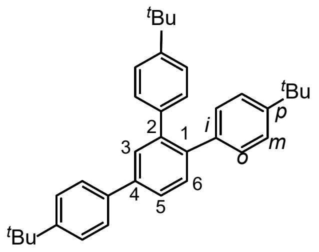

^{1}H NMR (500.13 MHz, C_6D_6, 25 ºC): δ = 7.85 (d, *J* = 2.0 Hz, 1H, H^3), 7.62 (dd, *J* = 7.9, 2.0 Hz, 1H, H^5), 7.60 (d, *J* = 8.4 Hz, 2H, Ar), 7.54 (d, *J* = 8.0, 2.0 Hz, 1H, H^6), 7.38 (d, *J* = 8.4 Hz, 2H, Ar), 7.33 (d, *J* = 8.4 Hz, 2H, Ar), 7.32 (d, *J* = 8.4 Hz, 2H, Ar), 7.17 (d, *J* = 8.5 Hz, 2H, Ar), 7.16 (d, *J* = 8.5 Hz, 2H, Ar), (s, 9H, Me), 1.173 (s, 9H, Me), 1.167 (s, 9H, Me); 1,3,5-isomer: δ = 7.96 (s, 3H, H^2), 7.65 (d, *J* = 8.3 Hz, 6H, Ar), 7.40 (d, *J* = 8.4 Hz, 6H, Ar), 1.29 (s, 27H, Me).

$^{13}C\{^1H\}$ NMR (125.8 MHz, C_6D_6, 25 ºC): δ = 150.4, 149.5, 149.4 (C^p), 141.5, 140.7, 139.8, 139.7, 139.2, and 138.5 (C^1, C^2, C^4, C^i), 131.9 (C^6), 130.2 (C^3), 130.1, 130.0, 127.3, 126.1, 125.3 (2C) ($C^{o,m}$), 126.3 (C^5), 34.6 and 34.5 (2C) (C^tBu), 31.5 and 31.4 (2C) (Me); 1,3,5-isomer: δ = 150.5 (C^p), 143.0 and 139.1 (C^1, C^i), 127.6, 126.1 ($C^{o,m}$), 125.3 (C^2), 34.6 (C^tBu), 31.5 (Me).

HR-APCI-MS (m/z): calcd for $C_{36}H_{42}$ $[M]^+$ 474.3286, found 474.3275 (err [mDa] = −1.1)

1,2,4-Tri-(2'-methylphenyl)benzene. Isolated yield = 68%

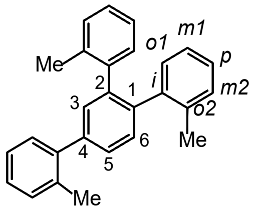

^{1}H NMR (500.13 MHz, C_6D_6, 80 ºC): δ = selected resonances for the 1,2,4-isomer: δ = 7.28 (d, *J* = 8.1 Hz, 1H, H^6), 7.27 (d, *J* = 1.6 Hz, 1H, H^3), 7.13 (m, 1H, H^5), 2.26 (s, 3H), 2.12 (s, 3H), and 2.11 (s, 3H) (Me); 1,3,5-isomer: δ = 7.14 (s, 1H, H^2), 2.24 (s, 3H, Me).

$^{13}C\{^1H\}$ NMR (125.8 MHz, C_6D_6, 25 ºC): δ = 142.5, 142.4, 142.2, 141.31, 141.30, and 140.0 (C^1, C^2, C^4, C^i), 135.9, 135.8, 135.6 (C^{o2}), 132.0 (C^6), 130.9 (C^3), 130.7, 130.31, 130.30, 130.24, 130.23, 129.0, 128.0, 127.7, 127.31, 127.30, 125.32, 125.29 ($C^{o1,m1,m2,p}$), 126.2 (C^5), 20.5 and 20.4 (2C) (Me); 1,3,5-isomer: δ = 142.2 and 141.3 (C^1, C^i), 135.6 (C^{o2}), 130.7, 130.2, 127.7, and 127.3 ($C^{o1,m1,m2,p}$), 126.2 (C^2), 20.5 (Me).

HR-APCI-MS (m/z): calcd for $C_{27}H_{24}$ $[M]^+$ 348.1873, found 348.1857 (err [mDa] = −1.6)

NMR spectra of tri-(aryl)benzenes

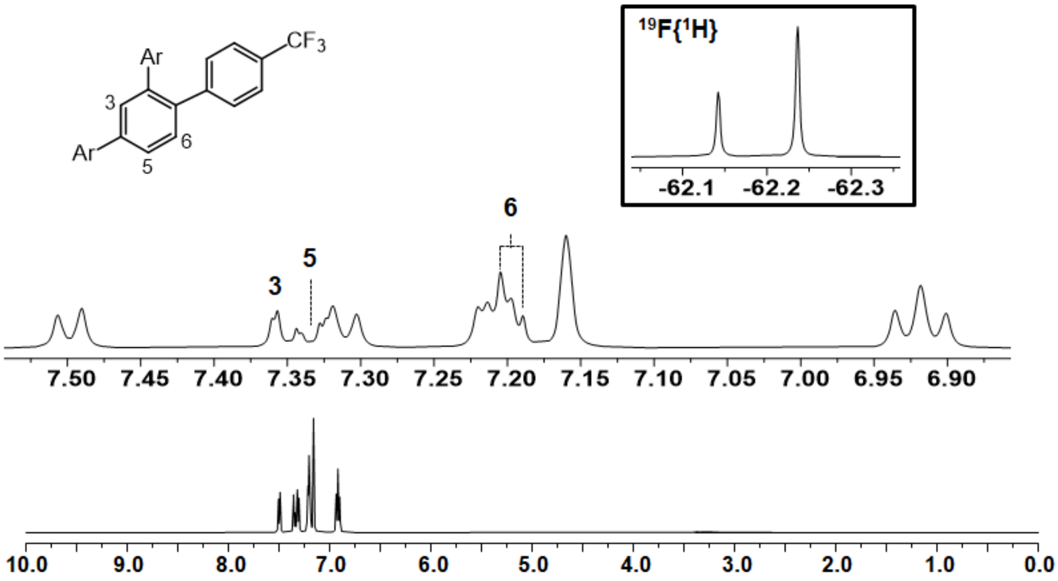

Figure S12. ^{1}H NMR spectrum of isolated tri-(4'-trifluoromethylphenyl)benzene in C_6D_6 at rt (bottom) and the corresponding aromatic region (top). The asterisk (*) denotes benzene. The inset shows the $^{19}F\{^{1}H\}$ NMR spectrum.

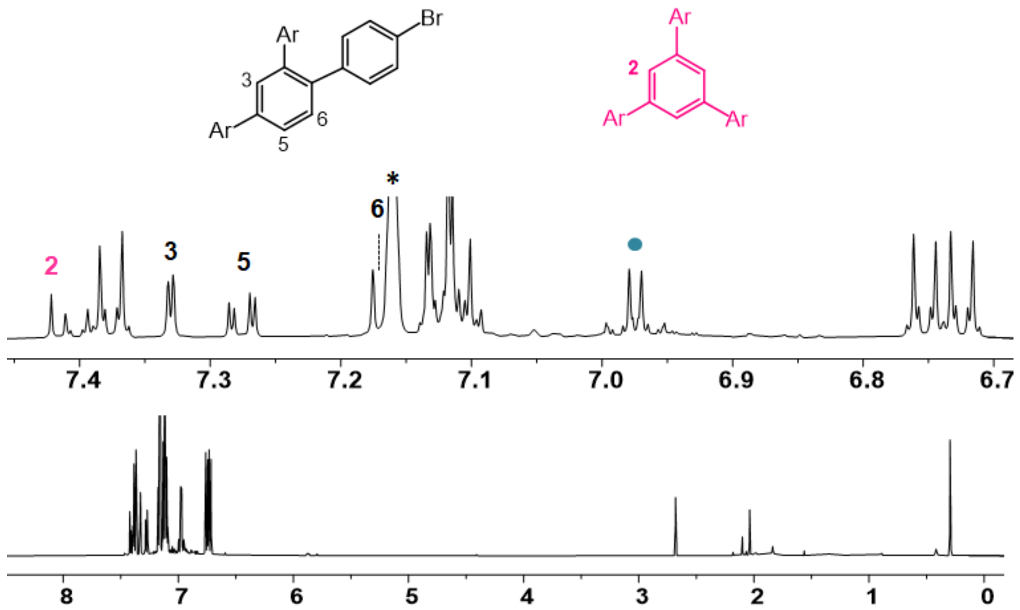

Figure S13. ^{1}H NMR spectrum of isolated tri-(4'-bromophenyl)benzene in C_6D_6 at rt (bottom) and the corresponding aromatic region (top). The asterisk (*) denotes benzene and the blue circle free alkyne.

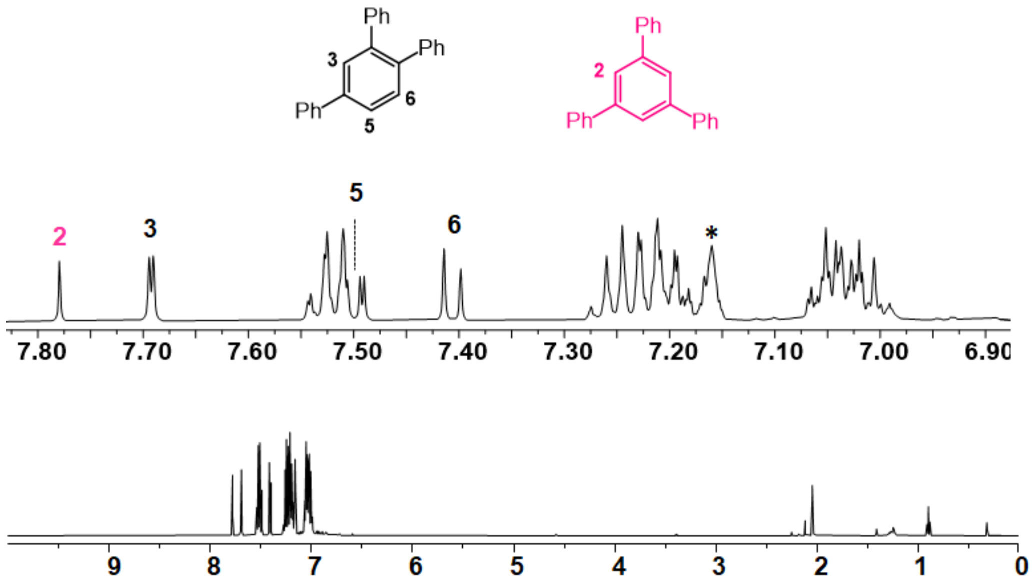

Figure S14. ^{1}H NMR spectrum of isolated tri-(phenyl)benzene in C_6D_6 at rt (bottom) and the corresponding aromatic region (top). The asterisk (∗) denotes benzene.

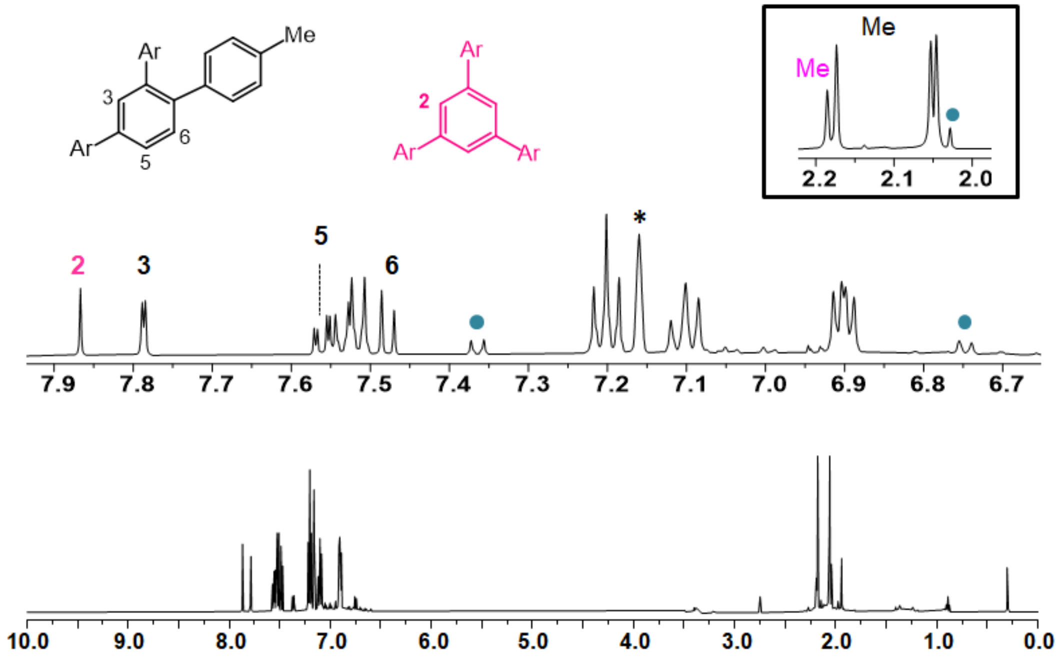

Figure S15. ^{1}H NMR spectrum of isolated tri-(4’-methylphenyl)benzene in C_6D_6 at rt (bottom) and the corresponding aromatic region (top). The asterisk (∗) denotes benzene and the blue circle free alkyne. The inset shows the methyl region.

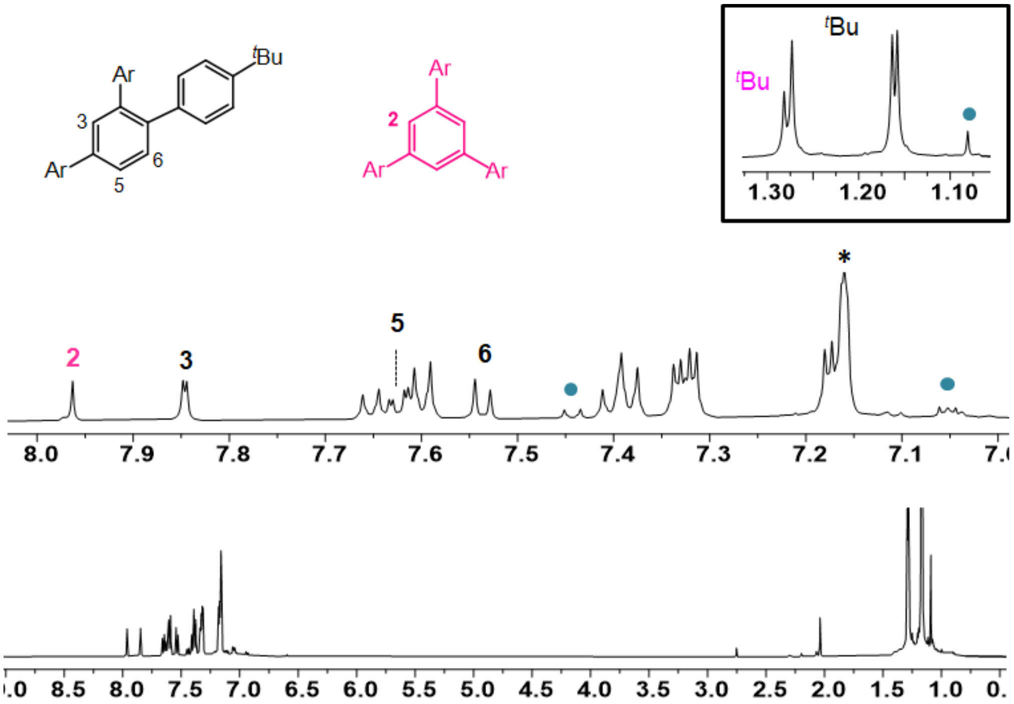

Figure S16. ^{1}H NMR spectrum of isolated tri-(4'-^{t}butylphenyl)benzene in C_6D_6 at rt (bottom) and the corresponding aromatic region (top). The asterisk (∗) denotes benzene and the blue circle free alkyne. The inset shows the ^{t}butyl region.

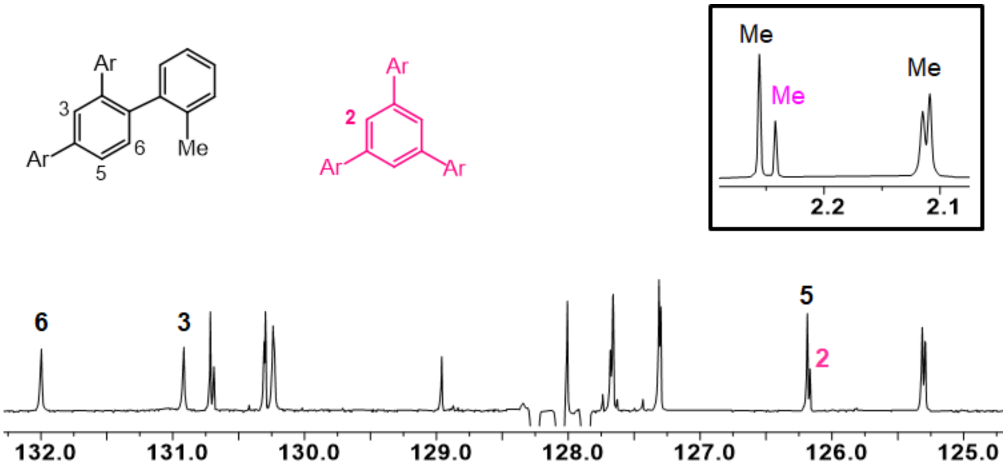

Figure S17. Selected region of the $^{13}C\{^{1}H\}$-apt NMR spectrum of isolated tri-(2'-methylphenyl)benzene in C_6D_6 at 80 ºC. The inset shows the methyl region of the ^{1}H NMR spectrum.

REFERENCES

(S1) Sharpe, H. R.; Geer, A. M.; Taylor, L. J.; Gridley, B. M.; Blundell, T. J.; Blake, A. J.; Davies, E. S.; Lewis, W.; McMaster, J.; Robinson, D.; Kays, D. L. Selective reduction and homologation of carbon monoxide by organometallic iron complexes**.** *Nat. Commun.* **2018**, *9,* 3757.
